# Legumes and Legume-Based Beverages Fermented with Lactic Acid Bacteria as a Potential Carrier of Probiotics and Prebiotics

**DOI:** 10.3390/microorganisms10010091

**Published:** 2021-12-31

**Authors:** Patrycja Cichońska, Małgorzata Ziarno

**Affiliations:** Department of Food Technology and Assessment, Institute of Food Science, Warsaw University of Life Sciences-SGGW (WULS-SGGW), 02-787 Warsaw, Poland; malgorzata_ziarno@sggw.edu.pl

**Keywords:** lactic acid bacteria, fermentation, legumes, plant-based beverages, legume-based beverages, bioactive metabolites, probiotics, prebiotics

## Abstract

Fermentation is widely used in the processing of dairy, meat, and plant products. Due to the growing popularity of plant diets and the health benefits of consuming fermented products, there has been growing interest in the fermentation of plant products and the selection of microorganisms suitable for this process. The review provides a brief overview of lactic acid bacteria (LAB) and their use in fermentation of legumes and legume-based beverages. Its scope also extends to prebiotic ingredients present in legumes and legume-based beverages that can support the growth of LAB. Legumes are a suitable matrix for the production of plant-based beverages, which are the most popular products among dairy alternatives. Legumes and legume-based beverages have been successfully fermented with LAB. Legumes are a natural source of ingredients with prebiotic properties, including oligosaccharides, resistant starch, polyphenols, and isoflavones. These compounds provide a broad range of important physiological benefits, including anti-inflammatory and immune regulation, as well as anti-cancer properties and metabolic regulation. The properties of legumes make it possible to use them to create synbiotic food, which is a source of probiotics and prebiotics.

## 1. Introduction

Fermentation is a natural food preservation method that has been used for centuries. Initially, this process was carried out spontaneously with microorganisms naturally present in food, whereas currently, selected starter cultures with specific compositions are used to this end [1]. The fermentation process is done by lowering the pH of the food, using microorganisms that convert the sugars present in the product into acid. It is most often accomplished with lactic acid bacteria (LAB), which produce lactic acid as the major product of carbohydrate metabolism [2,3,4,5,6]. Some microorganisms in this group also exhibit probiotic properties, and their consumption is beneficial to selected aspects of human health (e.g., gastrointestinal function, immunity) [7,8]. Fermentation is widely used in the processing of dairy, meat, and plant products [7,9]. In fermented foods, LAB shape the taste, texture, nutritional value, and produce of beneficial metabolites that affect the functional and healthy properties of food products [2]. Due to the growing popularity of plant diets and the health benefits of consuming fermented products, there has been growing interest in the fermentation of plant products and the selection of microorganisms suitable for this process. The expanding range of alternatives to dairy products has spurred new research into plant products as matrices for the production of fermented and probiotic products [10,11,12].

The global dairy alternatives market was valued at USD 20.50 billion in 2020 and is expected to expand at a compound annual growth rate (CAGR) of 12.5% from 2021 to 2028 [13]. A global survey conducted in 2019 showed that 40% of the surveyed consumers try to limit their consumption of animal proteins, which resulted from concerns about climate change [14]. Plant-based beverages are among the most popular dairy alternatives, usually treated as analogues of cow’s milk. The milk-based dairy alternatives product segment led the market and accounted for a revenue share of more than 67.0% in 2020 [13].

The main drivers of the plant-based beverage boom are the growing popularity of the plant-based diets and the growing concern for the environment among consumers [15,16]. Plant-based beverages are also consumed by people who are lactose intolerant, allergic to cow’s milk proteins, and interested in low-cholesterol foods [15,16]. With the growing number of consumers choosing alternatives to cow’s milk, the demand for non-dairy probiotic foods (both fermented and unfermented) is increasing. However, so far, probiotics have been used for enrichment mainly in dairy products due to their natural occurrence, properties, and widespread availability [17,18].

The most popular among the alternatives to cow’s milk are beverages made from soybeans, which are a type of legume [19,20]. However, there are also other legumes highly suitable for the production of plant-based beverages, e.g., beans, peas, and chickpeas [21]. Legumes are a source of carbohydrates, dietary fiber, and proteins, and they are rich in bioactive ingredients (including, e.g., polyphenols and phytosterols) [22]. Legumes are also a potentially suitable matrix for the fermentation process [23]. The development of legume-based beverages, subject to additional fermentation, can help expand and enrich the range of milk alternatives available [24]. With that in mind, the aim of this review was to evaluate legumes and legume-based beverages fermented with LAB as a potential carrier of probiotics and prebiotics. The review provides a brief overview of lactic acid bacteria (LAB) and their use in the fermentation of legumes and legume-based beverages. Its scope also extends to prebiotic ingredients present in legumes and legume-based beverages that can support the growth of LAB.

## 2. Lactic Acid Bacteria (LAB)

### 2.1. Characteristics of LAB

Lactic acid bacteria (LAB) are a phylogenetically heterogeneous group of Gram-positive bacteria that share metabolic and physiological characteristics [25]. These bacteria ferment carbohydrates into lactic acid (homofermentation), or into lactic acid, ethanol, and CO2 (heterofermentation) [7,9]. Sugars are fermented mainly under anaerobic conditions, but some species produce more lactic acid in the presence of a small amount of oxygen, e.g., *Lactobacillus delbrueckii* subsp. *bulgaricus*, *Lactiplantibacillus plantarum* subsp. *plantarum*, and *Leuconostoc mesenteroides*. They are non-sporulating cocci or bacilli, low in guanine and cytosine in chromosomal DNA (not more than 53 mole% of G + C pairs). LAB do not produce catalase, instead synthesizing superoxide dismutase, which removes reactive oxygen species [9,26,27,28]. The majority of LAB belongs to the genera *Lactobacillus, Leuconostoc, Pediococcus, Lactococcus, Streptococcus, Enterococcus, Carnobacterium, Aerococcus, Oenococcus, Tetragenococcus, Vagococcus*, and *Weissella* [7,9,25,27]. LAB are generally associated with the order of Lactobacillales (phylum Firmicutes); however, bacteria of the genus *Bifidobacterium* (phylum Actinobacteria) are often grouped together with them. *Bifidobacterium* are strictly anaerobic Gram-positive bacteria that also produce lactic acid as a major product of carbohydrate metabolism [2,29].

Lactic bacteria have the ability to regulate intracellular pH and thus survive in a relatively low pH environment [9]. LAB grow until the carbohydrates, amino acids, and other substances necessary for life are consumed in the medium; until toxic or growth inhibitory substances (e.g., hydrogen peroxide) accumulate; or until the concentration of hydrogen ions drops below the tolerance limits of the bacteria. For example, *Lactococcus lactis* subsp. *lactis* and *Streptococcus thermophilus* are grown in milk until the pH of the environment drops to 4.5, even if the nutrient stock is unlimited [3,9,30].

Due to the limited biosynthetic capacity of some compounds (called auxotrophy), LAB occur in environments rich in amino acids, vitamins, purines, and pyrimidines [9]. In laboratory conditions, LAB cultures are grown in media containing tryptone, yeast extract, and lactose. Lactose-free strains are cultured in glucose-containing media. Some of them are free living organisms, while others live in association with vertebrate animals and can also be found in humans and animals as potential pathogens (e.g., *Streptococcus pyogenes, S. pneumoniae*) [9,30]. Although most LAB are vitamin auxotrophs, some strains have the ability to synthesize water soluble vitamins, mainly B vitamins (including folates, riboflavin, and vitamin B12). For example, *Streptococcus thermophilus* and *Lactobacillus delbrueckii* subsp. *lactis* have the ability to produce folates, while *Lactococcus reuteri* have the ability to biosynthesize cobalamin [31].

LAB play an important role in many industrial sectors, including agricultural, food, and clinical [7,9]. In nature, these microorganisms occur in fermented dairy, meat, and vegetable products, in the digestive and urogenital tracts of humans and animals, as well as in soil and water [7,9,25,27]. LAB are generally considered safe for human consumption. Most of them are classified as GRAS (generally recognized as safe). Currently, the most common use of LAB is as starter cultures for the production of fermented foods. Some LAB have been classified as probiotic [2]. In addition, they are widely used in the industrial production of lactic acid, polyols, vitamins, and food ingredients due to their resistance to environmental stress and their versatile metabolic properties. In fermented foods, LAB shape the taste, texture, and nutritional value of products, mainly by performing glycolysis (sugar fermentation), lipolysis (fat breakdown), and proteolysis (protein breakdown) [2,28]. Their use as starter cultures is motivated not only by their production of lactic acid, but also the production of beneficial metabolites that affect the taste and properties of food products, including organic acids, alcohols, aldehydes, esters, sulfur compounds, polyols, exopolysaccharides, and antimicrobial compounds. The starter cultures used in the food industry most often include LAB from the genera *Lactococcus, Streptococcus, Lactobacillus, Pediococcus*, and *Leuconostoc* [2,32].

### 2.2. Fermentation with LAB

LAB are widely used in the fermentation of animal and plant products [9,18,33,34]. Their main application is in the fermentation of dairy products (e.g., yoghurt, kefir, buttermilk, koumiss, cheese) [35,36], meat [37], fish [38], fruit [39], vegetables [40], and cereal products [41]. LAB fermentation is one of the main forms of food preservation that does not require the use of chemical food additives [7]. LAB lower the pH of food, inhibit the growth of putrefactive bacteria, improve the organoleptic properties of food, enhance the nutrient profile, and optimize health benefits. LAB enzymes (including amylases, proteases, and lipases) hydrolyze polysaccharides, proteins, and lipids into flavors, fragrances, and texture modifiers and confer properties attractive to consumers [18,42].

LAB grow in the temperature range of 10 to 45 °C, salinity of 6.5% NaCl, and pH between 4 and 9.6. The fastest LAB-mediated fermentation and biomass production processes take place at a temperature of 26 to 42 °C, whereas the bacteria themselves are mostly grown at a temperature of 35–37 °C [9]. LAB can be classified as homofermentative or heterofermentative organisms, based on their ability to ferment carbohydrates. Homofermentative LAB (e.g., *Lactococcus, Streptococcus*) use the EM glucose degradation pathway (Embden–Meyerhof) and produce two lactate molecules from one glucose molecule. Heterofermentative bacteria (e.g., *Leuconostoc, Wiessella*) catabolize glucose along the 4PP (pentose phosphate) pathway and produce lactate, ethanol, and carbon dioxide from a single glucose molecule [7,9,25].

Traditionally, fermentation was carried out mainly by inoculating the food with a sample of a previously fermented product, which is known as backslopping. However, the products obtained via this process were of heterogeneous quality [2]. Currently, LAB are predominantly added to products in the form of commercial, freeze-dried starter cultures, which enables the production of homogeneous, high quality products on a large scale. The starter cultures are divided into defined- and mixed-strain cultures. Strain-defined cultures are pure cultures with known physiological and technological characteristics. They consist of two to six strains, used in rotation either as paired single strains or as multiple strains, and can be used to make high quality products on an industrial scale. Mixed strain cultures contain an unknown number of strains of the same species and may also contain bacteria from different LAB species or genera [2]. The most common use of starter cultures is in dairy production. The bacteria associated with fermented milk products are the genera *Lactobacillus, Lactococcus, Leuconostoc, Pediococcus, Bacillus, Propionibacterium,* and *Bifidobacterium*. In the production of fermented dairy products, LAB are used as primary starter cultures involved in the acidification of the product (e.g., *Lactobacillus* sp., *Leuconostoc* sp., *Streptococcus* sp.) and also as secondary starter cultures responsible for shaping aroma, taste, and texture (e.g., *Propionibacterium* sp., *Brevibacterium* sp., *Debaryomyces* sp., *Geotrichum* sp., *Penicillium* sp., *Enterococcus* sp.) [7,42].

In addition to the ability to bio-conserve food, LAB can also synthesize other substances that affect the organoleptic characteristics of food and its properties [7,32]. LAB require essential amino acids and peptides to grow, which are released in the process of proteolysis of proteins present in the raw material. They are capable of degrading the fats in dairy products, turning them into methyl ketones, lactones, thioesters, and hydroxy acids, which give dairy products their ultimate flavor. The products of these conversions help develop the characteristic organoleptic features of fermented products [7,9,32]. The fermentation process has also been found to elicit a beneficial effect on reducing the content of anti-nutritional factors (ANFs), e.g., phytates and protease inhibitors, in plant products [18]. In addition, LAB have the ability to produce antimicrobial substances called bacteriocins, which are ribosome-synthesized peptides that exhibit antimicrobial activity, can inhibit pathogenic microflora in food [7,43,44], and are usually secreted outside the bacterial cell. Bacteriocins synthesized by LAB strains, including those of the genera *Lactococcus* sp., *Lactobacillus* sp., *Pediococcus* sp., *Carnobacterium* sp., or *Leuconostoc* sp., are considered safe to be used in food production [32,45,46].

LAB also include bacteria recognized as probiotic [8]. Probiotics were originally defined as microorganisms that promoted the growth of other microorganisms [8,29]. In recent years, they have been more precisely defined as mono- or mixed cultures of living microorganisms which, when administered to an animal or a human, are beneficial for the host, improving the properties of the native microflora. In the context of food, probiotics are defined as preparations or dietary supplements that improve human and animal health [8,29]. Their function is to restore the natural state of the human microflora that has been disturbed by improper nutrition, disease, or the healing process. The main purpose of consuming probiotics—whether as food and pharmaceutical preparations—is for their beneficial effect on the colonic microflora [18]. Probiotics can be consumed in fermented foods or in the form of capsules, pills, and tablets [17]. In order for a probiotic strain to be considered for human use, it should be isolated from the human microflora, which grants it a high capacity for adhesion to the human intestinal cell walls. The strain must also be safe and not pose a threat to the host. The probiotic microorganisms most extensively isolated from fermented foods and from the animal/human digestive systems include *Lactobacillus, Pediococcus, Bifidobacterium, Lactococcus, Streptococcus*, and *Leuconostoc* [7,47].

Various mechanisms underlying the action of probiotics have been considered. In general, probiotics elicit a positive effect on the human body, e.g., by competing with pathogenic bacteria to bind to the cells of the intestinal epithelium; enhancing the function of the intestinal epithelial barrier; inhibiting the growth of pathogens by secreting antimicrobial peptides; stimulating the production of immunoglobulins; enhancing phagocytosis; increasing the activity of NK (Natural Killer) cells; promoting cellular immunity against pathogens; and preventing inflammation [7]. In addition to lactic acid, probiotic LAB produce several bioactive metabolites (antimicrobial and shelf-life extending), such as organic acids, short-chain fatty acids, carbohydrates, antimicrobial peptides, enzymes, vitamins, cofactors, or immune signaling compounds—these substances are also known as postbiotics [48].

Consuming probiotics is beneficial to many aspects of health, especially in the prevention and treatment of infections and gastrointestinal diseases. The therapeutic uses of probiotics also include the prevention of genitourinary diseases, constipation relief, protection against traveler’s diarrhea, management of hypercholesterolemia, protection against colon and bladder cancer, as well as prevention of osteoporosis and food allergies [7,8,49]. Additionally, the consumption of probiotic bacteria prevents obesity, type II diabetes and cardiovascular diseases, while also stimulating the immune system. Probiotics help relieve the symptoms of lactose intolerance by producing the enzyme galactosidase (lactase), which breaks down lactose into simple sugars [7,8,29,49,50]. The health benefits of consuming probiotic LAB may result from the combination of viable microorganisms contained in fermented foods and bioactive ingredients released into the food as by-products of the fermentation process [51]. Healthy benefits of foods fermented with probiotic LAB are summarized in Table 1.

## 3. Characteristics of Legumes

Legumes are a staple food in many countries around the world. The most commonly eaten varieties are beans, faba beans, chickpeas, peas, lentils, cowpeas, lupins, and soybeans [62,63,64]. Legumes are suitable for growing under adverse environmental conditions and in a variety of growing systems due to their low input requirements, short growing season, and nitrogen fixation capacity [64]. As shown in Table 2, legumes are a rich source of carbohydrates (30–60% of total content), dietary fiber (9–25%), and protein (19–36%) containing the necessary amino acids such as lysine, leucine, and arginine [22,65,66]. The carbohydrates include monosaccharides, oligosaccharides, other polysaccharides, and starch. In legumes, starch is the main source of available carbohydrates (22–45% of total content) along with oligosaccharides (1.8–18%) and dietary fiber (4.3–25%). Legumes are usually low in fat and contain no cholesterol, with a favorable fatty acid profile dominated by unsaturated fatty acids (Table 2). They are also a good source of iron, calcium, zinc, selenium, magnesium, phosphorus, copper, potassium, and B-group vitamins; however, they are poor in vitamin C and fat-soluble vitamins. The moisture content of all dry legumes varies between 9 and 3%, which makes them suitable for long-term storage [64,67].

Legumes contain bioactive substances that play a significant metabolic role in the human body. Their action can be considered positive, negative, or in some cases, both. Dietary fiber, resistant starch, polyphenols, and phytosterols are referred to as health-promoting ingredients [63,68,69]. Legumes are rich in both soluble and insoluble fiber. The seed coat is rich in water-insoluble polysaccharides, while the cotyledon fiber consists of hemicelluloses, pectin, and cellulose with varying degrees of solubility. Resistant starch is a non-digestible fraction of starch, which, after passing into the large intestine, functions physiologically similarly to dietary fiber [68,70]. Consuming fiber as part of one’s daily diet is essential for nominal intestine function, which is implied in lowering the risk of development of many chronic diseases, including colon cancer, heart disease, and diabetes [68,70]. Resistant starch and fiber pass through the stomach and small intestine in undigested form until they reach the colon, where they play a prebiotic role. Fiber and resistant starch also help reduce body weight, increase stool volume, and decrease colon pH, while also lowering serum cholesterol and triglycerides. In addition, they reduce the glycemic index of legumes, regulating postprandial glycemia and insulin sensitivity [64,68,70]. 

The polyphenols present in legumes are bioactive compounds that have a broad therapeutic potential due to their antioxidant activity. They work by delaying or preventing the oxidation of lipids, proteins, and DNA by reactive oxygen species [68,71]. The amino acids of legume proteins (mainly tyrosine, phenylalanine, tryptophan, and cysteine) also exhibit antioxidant properties that result from their ability to donate protons to free radicals [72]. Epidemiological evidence shows that dietary intake of legume antioxidants provides a protective effect against certain chronic diseases associated with oxidative stress such as cardiovascular disease, cancer, obesity, diabetes, and hypercholesterolemia [67,68,72]. 

Phytosterols are plant sterols with a structure similar to cholesterol. These compounds belong to the group of steroid alcohols that occur naturally in legumes. As a natural component of plant structures, phytosterols contribute to the regulation of the fluidity and permeability of cell membranes [73,74]. The most common phytosterols are β-sitosterol, campesterol, and stigmasterol [73,74]. Phytosterols are well known for a wide range of health benefits, the most important of which are lowering blood LDL cholesterol and reducing its absorption in the intestine [64,68]. 

Certain bioactive substances of legumes have been found to be ANFs. Some of them play an important role in the mechanisms used by plants to protect themselves against predators or environmental conditions, while others are storage compounds, accumulated in seeds as an energy reserve [62,75,76]. The major ANFs of legumes include oligosaccharides from the raffinose family, protease inhibitors, phytates, and saponins. These factors, apart from their negative impact on the body, often also have a positive and health-promoting effect, which has provoked some debate as to whether it is necessary to reduce their content in legumes [62,75]. 

Oligosaccharides are carbohydrates from the raffinose family, including, i.e., raffinose, stachyose, and verbascose [62,68]. They are well known as ANFs that cause gas and discomfort upon consumption of legumes as a result of α-galactosidase depletion in the human body [62,77]. When ingested, they pass into the large intestine, where they are anaerobically fermented by intestinal bacteria to produce hydrogen, carbon dioxide, and methane [75]. However, these oligosaccharides are also considered a component of dietary fiber and may show prebiotic effects by stimulating the growth of *Lactobacilli* and *Bifidobacteria* beneficial for the intestines and limiting the development of putrefactive *Enterobacteria* in the colon [62,68,75]. 

Protease inhibitors in legumes inhibit the action of digestive enzymes, such as trypsin and chymotrypsin, through competitive binding [22,75]. These inhibitors can reduce protein digestibility and nutrient absorption. On the other hand, they are considered to be natural bioactive substances that may have anti-cancer effects [62,75,78].

Phytates are salts of phytic acid, which, when consumed in tandem with legumes, may reduce the bioavailability of minerals and limit enzymatic digestion of starch and proteins [62,64]. Excess phytic acid forms insoluble complexes with copper, zinc, iron, and calcium, limiting their availability in the intestine [64,78]. However, there are some beneficial effects of phytic acid, such as reducing bioavailability and toxicity of heavy metals (e.g., cadmium and lead) present in food. Phytates also elicit antioxidant, anti-cancer, and DNA-protective effects [64,75]. 

Saponins are irritating to the stomach and can also inhibit the absorption of nutrients in the intestine [64,75]. The potential benefits of consuming saponins include reduced risk of development of cardiovascular disease and certain cancers. Some research suggests that saponins may lower cholesterol by forming insoluble complexes with it in the intestine [75].

ANFs in legumes are usually removed by applying a number of technological treatments such as soaking, microwaving, extrusion, steaming, boiling, roasting, infrared, starch modification, germination, and fermentation [22,69,75,76]. These treatments also improve the taste of these raw materials, increase the bioavailability of nutrients, and increase the digestibility and absorption of starch and protein [64,75]. Modification of starch (total or partial gelatinization) not only contributes to reducing the content of ANFs in legumes but also affects the technological properties by improving emulsifying, foaming, water retention capacity, and thickening [79,80]. Some of the ANFs in legumes are reduced during initial processing—heat treatment and soaking of the raw materials. Soaking partially reduces the oligosaccharide content. Higher levels of oligosaccharides have been demonstrated in legumes that have not been soaked before the heat treatment [77]. For example, in lentils, heat treatment almost completely eliminates the trypsin inhibitors—tannins and phytic acid. However, it also reduces the content of minerals and some amino acids (lysine, tryptophan, and sulfur-containing amino acids) [81]. Some of the ANFs (e.g., tannins and saponins) are thermally stable, but their content can be reduced by sprouting and/or fermentation [75]. Fermentation reduces the content of thermostable ANFs in legumes, which leads directly to an increase in digestibility of proteins and bioavailability of certain nutrients [77,78].

## 4. Fermentation of Legumes Using LAB

Fermented legumes have been consumed by humans for centuries [82]. Currently, legume fermentation is widely used in the production of intermediate bakery products (e.g., cookies, pasta, bread), ingredients for Asian cuisine, substitutes for dairy products, and as an ingredient of animal feed [83,84,85,86]. The addition of fermented legumes to food products may improve their technological properties and nutritional value, e.g., by increasing the protein digestibility and mineral availability, reducing the ANFs content, and improving the viscosity of the final product [87,88,89,90,91,92,93,94,95,96,97,98]. The use of legumes fermented with LAB as ingredients of various types of food is summarized in Table 3.

LAB often represent the indigenous microflora of legumes. Therefore, in the production of fermented legumes, fermentation is induced not only by inoculation of raw material with LAB but also through spontaneous fermentation [84]. The use of LAB induces changes in the organoleptic, functional, and technological properties of legumes. The type and extent of these changes depend on the raw material, the bacterial species used, and the fermentation parameters. LAB are characterized by a variety of pathways to produce acids and other metabolites [99]. The fermentation of plant matrices is dependent upon their ability to adapt rapidly and metabolize the available nutrients. Adaptation is species- and strain-specific, as well as determined by the plant material. This is due to the diversity of plant environments and inherent chemical/physical parameters, such as phenols, fermentable carbohydrates, and environmental pH, which determine whether conditions are optimal for bacterial growth. The same microorganisms can behave differently in plant and animal matrices. Not all strains are endowed with an optimal portfolio of enzymes and metabolic traits, and therefore it is necessary to properly adapt the plant matrix and microorganisms for fermentation [100].

The selection of suitable microorganisms is based on whether they improve or impair the properties of the raw material. Poorly chosen microorganisms may negatively affect functional properties, e.g., by reducing protein solubility and emulsifying capacity [99]. Strain-specific metabolic features of the LAB, in synergy with the activity of plant enzymes, can improve the bioavailability and bioactivity of phytochemicals while also significantly boosting functional microbial metabolites, with beneficial consequences for human health [100,101,102]. Landete et al. (2015) [103] fermented flours from yellow soy beans and Mung beans. Fermentation with *Lactiplantibacillus plantarum* subsp. *plantarum* promoted bioactivity due to conversion of glycosylated isoflavones into bioactive aglycones. Moreover, an increase in the content of the bioactive vitexin tryptophan was observed [103].

In the production of fermented foods, optimization of the process parameters is crucial. Excessive fermentation time may result in compounds with undesirable properties. For example, fermenting pea protein isolate for 24 h yielded more acceptable products compared to the 48 h fermented samples [99]. Longer fermentation time induced the production of metabolites unappealing to consumers. In another study, by Shi et al. (2021) [104], the fermentation parameters influenced the properties of fermented legumes. The soluble protein levels in pea protein isolate (PPI) decreased at longer fermentation times, and the protein profile changed after 15 h of fermentation. A 10-hour fermentation process was found to be optimal for *Lactiplantibacillus plantarum* subsp. *plantarum* in terms of removing off-flavor while maintaining protein functionality. About 42% of the aldehydes and 64% of the ketones were removed and a small amount of alcohol was produced. This change in the aroma profile was found desirable for PPI products that would be used in the production of yoghurt substitutes [104].

Legumes subjected to fermentation are often of higher nutritional value than unfermented seeds [82]. Legume fermentation may improve protein digestibility and related nutritional values while increasing the biological availability of total fiber and phenols [84,105]. Fermentation can increase the levels of vitamins, amino acids, minerals, and short-chain fatty acids [106]. The improvement in the nutritional profile of legumes upon LAB fermentation is mainly due to the release of amino acids and bioactive compounds, reducing the amount of ANFs through direct (activation of microbial enzymes) and indirect action (activation of endogenous enzymes), while also enhancing in vitro protein digestibility and antioxidant potential [107,108].

Soybean fermentation by *Lactiplantibacillus plantarum* subsp. *plantarum* has been shown to improve protein digestibility and the total phenolic content by the end of fermentation [109]. In addition to increasing the nutritional value of legumes, fermentation also facilitates biological preservation of food by action of the various antimicrobial compounds produced by LAB, including bacteriocins, H_2_O_2_, CO_2_, and organic acids that inhibit the growth of harmful microorganisms [106,110].

Consumption of selected fermented legumes has been linked to a reduction in the incidence and severity of chronic diseases such as cardiovascular disease, breast and prostate cancer, menopausal symptoms, and bone loss [82,111,112,113,114]. Fermentation of bean extracts with *Lactiplantibacillus plantarum* subsp. *plantarum* may have a potential antihypertensive effect due to the high content of GABA (gamma-aminobutyric acid) and the activity of ACEI (angiotensin converting enzyme) [114]. Likewise, fermentation of pea seeds with *Lactiplantibacillus plantarum* subsp. *plantarum* resulted in potentially antihypertensive peptides being released during in vitro digestion [115]. Sweet lupine flour fermented with *Lactiplantibacillus plantarum* subsp. *plantarum* and *Limosilactobacillus reuteri* has shown noticeable antiproliferative activity against Caco-2 and MCF-7 cancer cell lines [116].

Fermentation increases the content of antioxidant components such as phenolic compounds, peptides, protein derivatives, and vitamins that are released or produced by a complex microbial enzyme system [117,118,119]. The metabolic activity of microorganisms varies across species and strains, which is why the exact increase in antioxidant activity in the fermented material is determined by the starter culture used [118]. Polyphenols are the main natural antioxidants present in food; however, they are often bound to the cell wall, glycosylated or in polymeric form, which affects their bioavailability [118]. The metabolic activity of LAB may induce their release or conversion into more active forms. Fermentation also influences the bioavailability of other compounds, such as vitamins and exopolysaccharides, further enhancing their antioxidant activity in vitro and in vivo [100,118].

The main limitation in the development and consumption of legume-based foods lies in the ANFs contained within [106]. The presence of these factors leads to reduced digestibility of proteins and bioavailability of certain nutrients [83,120]. Legumes contain heterogeneous and species-dependent ANFs, such as oligosaccharides, phytic acid, tannins, alkaloids, lectins, pyrimidine glycosides (e.g., vicin and convicin), and protease inhibitors [84]. ANFs reduce the digestibility and bioavailability of other nutrients as well. Some of them are thermolabile (e.g., protease inhibitors and lectins) and easily removed by heat treatment. Others (e.g., phytic acid, raffinose, tannins, and saponins) remain unchanged by heating. Thermostable components can be removed efficiently using biological methods (including fermentation) [83,84]. 

Fermented soy products have a low content of trypsin inhibitors and phytates [121]. Fermentation of mung beans and soybeans with *Lactobacillus delbrueckii* subsp. *bulgaricus, Lb. acidophilus*, and *Lacticaseibacillus casei* has been found to reduce the content of phytic acid compared to unfermented seeds [122]. Red kidney beans fermentation with *Lactobacillus acidophilus, Bifidobacterium,* and *Streptococcus thermophilus* results in reduction of phytates, trypsin inhibitor activity, saponins, tannins, and raffinose oligosaccharides [123]. Faba bean seeds fermented with *Lactiplantibacillus plantarum* subsp. *plantarum* have shown reduced trypsin inhibitor activity, condensation of tannins, and better in vitro protein digestibility [124].

LAB-fermented products may affect the sensory perception of legume products [125]. LAB produce different types of metabolites during the fermentation of proteins, carbohydrates, and lipids, including diacetyl, acetoin, ethyl acetate, and ethyl propanoate, which impart flavor and aroma to the product. LAB produce various organic acids, such as acetic acid, lactic acid, and propionic acid, during the metabolism of food ingredients that impart the product a sour taste. Organic acids and such substances as alcohol and aldehydes react to form various flavor compounds that enhance the taste of the fermented product [106,125]. Pea protein products fermented with LAB starter culture and yeast have been shown to incite better sensory perception than unfermented pea protein products. Most of the molecules responsible for the unpleasant notes of legumes have been found to degrade during the fermentation process [126]. The effect of fermentation by LAB on selected properties of legumes is summarized in Table 4.

## 5. Legumes as Raw Materials for the Production of Fermented Plant-Based Beverages

### 5.1. Characteristics of Plant-Based Beverages

Plant-based beverages are products that do not have a specific definition and classification in the literature. They are most often categorized as products obtained by water extraction of shredded plant materials, in the form of colloidal suspensions or emulsions [19,127,128]. Plant-based beverages are made from a variety of raw materials:

- cereals (e.g., oats, rice, millet, spelled);
- legumes (e.g., soybean, lupine, peas);
- nuts (e.g., hazelnuts, pistachios, almonds, walnuts);
- seeds (e.g., sesame, flax, hemp, sunflower);
- pseudocereals (e.g., quinoa, amaranth, buckwheat);
- other materials (e.g., coconut) [19,127,129,130].

Plant-based beverages are becoming more and more popular every year [19]. This has been prompted, in large part, by the shift away from cow’s milk and its derivatives among consumers, which is spurred by multiple factors. The most common motivator behind this trend is allergy and/or intolerance to milk components, especially lactose and casein [19,127,131]. The main consumers of plant-based beverages are vegetarians and vegans, who avoid animal products for ethical reasons and prefer plant-based beverages for their health benefits. Their popularity is further driven by the growing environmental awareness of consumers [16,132,133,134]. Intensive animal farming leads to depletion of water resources and high greenhouse gas emissions, which is why reining in growth of this sector should be prioritized in environmental governance [135,136].

Plant-based milk substitutes are usually designed to have a similar appearance, taste, and shelf life to cow’s milk, so that they can be used in a similar manner. In practice, however, each type of plant-based beverage has its own unique properties, which are a function of their composition and the unit operations involved in their production [15].

**Figure 1:**
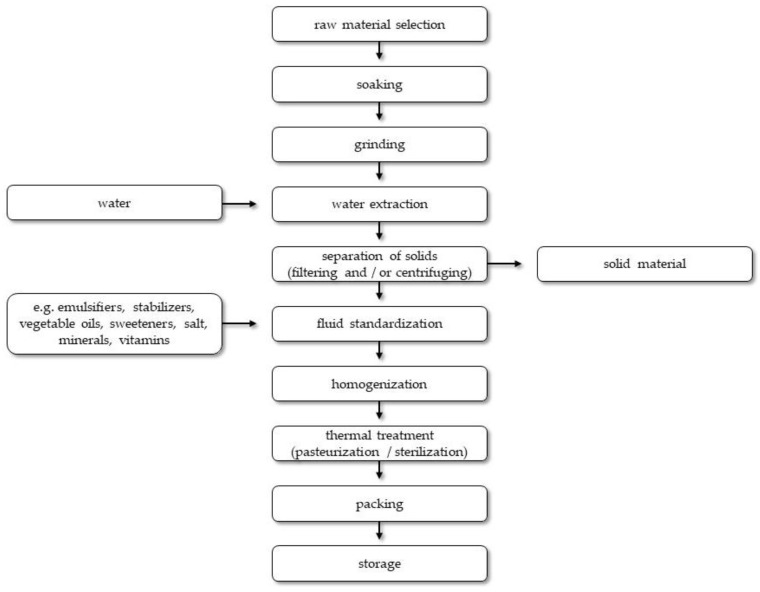
The general production technology of plant-based beverages.

The production technology of each plant-based beverage is tailored to the raw materials used, but the general outline of the process is usually the same [131]. Initially, raw materials are inspected and cleaned to prepare them for further processing. They are then subject to the process of soaking, grinding, and water extraction. The resulting slurry is filtered and/or centrifuged to remove solid matter. The filtered fluid is subject to standardization processes wherein the composition of the beverages is unified, and various types of ingredients are added to improve the technological and functional properties of the product [127,131]. Emulsifiers and stabilizers are added to increase the stability of the product (e.g., cellulose, tapioca, gella, guar, carrageenan, locust bean gum, lecithin). The beverages are usually amended with vegetable oils (such as peanut or sunflower oil) and sweeteners (such as sugar, agave syrup, fructose, and maltodextrin). Salt is added to shape palatability. Additionally, the plant-based beverages are fortified with calcium and vitamins (e.g., A and D) to make their nutritional value more similar to cow’s milk, then homogenized to reduce the particle size and unify the structure. The resultant beverage is heat treated (pasteurized or sterilized) in order to improve the stability of the suspension and inactivate microorganisms [127,129,131,137]. An overview of the general plant-based beverages production technology is shown in Figure 1. 

Plant-based beverages are complex media that contain small particles (e.g., fat droplets, protein particles, or plant cell fragments) dispersed in an aqueous medium [15]. The characteristics of these colloidal particles (i.e., their composition, structure, size, interfacial properties, and interactions) ultimately determine the physical, functional, sensory, and nutritional properties of plant-based milk substitutes. They determine the appearance, texture, taste, and stability of the drink, as well as bioavailability of individual nutrients. The characteristics of the particles contained in plant-based beverages and their stability are often determined by the type of processes used in their production technology and the storage conditions [15,19].

The most frequently consumed plant-based beverages include soy, almond, coconut, oat, and rice beverages [15,127,138]. The growing popularity of such products has prompted producers to employ a wider range of raw materials in making their products [131]. Categorizing cow’s milk substitutes is an oft-raised issue. They are often referred to as “plant milks”, but this is a misnomer as they do not meet the definition and do not have the nutritional value of milk [20,139]. Commission Regulation (EU) No 605/2010 of 2 July 2010 laying down animal and public health and veterinary certification conditions for the introduction into the European Union of raw milk and dairy products intended for human consumption defines “raw milk” as milk produced by the secretion of the mammary gland of farmed animals [140]. Naming plant-based beverages as milk is therefore a generalization that may mislead consumers.

The composition and properties of milk and plant-based beverages differ on several levels. The nutritional value of plant-based beverages varies depending on the raw material from which they are produced and the production technology used [16]. Nevertheless, in most cases they are high in carbohydrates and low in protein, containing up to 30 times less protein than cow’s milk. Soy beverages have the most similar protein content to cow’s milk [16,141]. Even so, vegetable protein is inferior, mainly due to the presence of limiting amino acids (lysine in cereals, methionine in legumes) and poor digestibility [127,129]. In most cases, plant-based beverages are low in fat unless supplemented with vegetable oils. Compared to milk, plant-based beverages have a low content of saturated fatty acids (less than 0.7 g/100 g), with the exception of coconut beverage, which is SFA-rich (about 1.7 g/100 g) [137]. Plant-based beverages are dominated by unsaturated fatty acids, mainly in the form of oleic, linolenic, and linoleic acids [127,137,141]. Cow’s milk is a natural source of calcium (120 mg/100 g on average), and plant-based beverages tend to be low in this nutrient if not fortified during production. Cow’s milk contains naturally-occurring vitamins A and trace amounts of vitamins D, E, K, C, and B. It is also a source of phosphorus, potassium, zinc, and easily digestible magnesium, as well as small amounts of sodium and iron [129]. 

Table 5 shows the nutritional value of selected plant-based beverages. The values for the individual nutrients are presented as a range of values due to the discrepancies between literature sources. This variability is mostly attributable to different authors using different technologies for the production of plant-based beverages.

Plant-based beverages, compared to cow’s milk, have a nutritious fatty acid profile due to the low content of saturated fatty acids and the dominant share of unsaturated fatty acids in total fat [165,166]. In addition, plant-based beverages contain bioactive ingredients with health-promoting effects, such as β-glucans (present, e.g., in oat beverages), phytosterols, and polyphenols (present, e.g., in soy and almond beverages) [131,137,141]. These products do not contain lactose, which cannot be consumed by people allergic to this component, and cholesterol, which is often avoided by people with hypercholesterolemia [16]. Scientific reports indicate that individual components of plant-based beverages have a positive effect on health. Unsaturated fatty acids and phytosterols in such products provide health benefits, including reducing the risk of heart disease, stroke, and heart attack, as well as lowering cholesterol, preventing cancer, modulating the immune system, and slowing aging [167,168]. In addition, plant-based beverages are rich in antioxidants, the consumption of which can prevent cancer of the ovary, breast, stomach, prostate, and lung, mainly by reducing oxidative stress in the body [169,170].

### 5.2. Legume-Based Beverages

From a nutritional point of view, legume seeds are high in protein, vitamins, and minerals [171]. Of all plant-based beverages, legume-based beverages have the most balanced composition while also having a low glycemic index [152]. Their protein fraction is around 3.0–4.0 %, similar to cow’s milk (i.e., 3.3–3.5%), while other types of cereal- and nut-based beverages usually have a protein fraction of between 0.1% and 1.0% [152,171].

So far, soybeans are the most commonly used to produce plant-based beverages and the most widely described in the literature among the group of legumes. Though soybean beverages are mainly produced on an industrial scale, other types of legumes may also be a suitable matrix for the production of plant-based beverages [21]. In the case of beverages from legumes other than soybean, most of the production technologies have not yet been entirely refined, whereas their composition and properties are variable, directly determined by the technological processes applied [172].

Generally, plant beverages are typically an oil-in-water (O/W) emulsion with water as the aqueous phase and oil as the dispersed phase. These two phases are immiscible and thermodynamically unstable, tending to separate into fractions and aggregate particles as a result [173]. Legume-based beverages are a complex colloidal system formed by dispersed particles such as proteins, oil droplets, and solids from raw materials. These factors make it difficult to obtain stable products [152,173]. Legume proteins exhibit emulsifying properties, which result from their ability to adsorb at the oil–water interface and form stabilizing protein films around the oil droplets. Such properties have been identified in the proteins of soybean, chickpeas, peas, and beans and indicated them to be plant proteins that may potentially affect the stabilization of O/W emulsions [171,174]. 

The basic technology for the production of beverages from legume seeds includes the following stages: raw material selection, soaking, grinding, water hydrolysis, filtration, homogenization, and thermal treatment [21,152]. Apart from technological measures, the process of making legume-based beverages also employs sweeteners, salt, aromas, and other food additives, as dictated by the local market. These treatments are used to increase the overall acceptance of beverages [175]. 

In the production of vegetable beverages from legumes, it is vital to include processes designed to reduce the content of anti-nutrients and increase the digestibility of proteins. For example, ingredients such as trypsin inhibitors, lectins, and hemagglutinins inhibit the action of proteolytic enzymes, resulting in reduced digestibility and absorption of proteins [175]. The processes that directly reduce ANFs include, among others, soaking, cooking, enzymatic treatment, protein extraction, germination, and fermentation [172,176]. The use of enzymatic treatment, fermentation, and germination processes increases the bioactivity and bioavailability of phenolic compounds, while activating the release of bioactive peptides. Such treatments make it possible to obtain functional food products or beverages with improved health-promoting properties [172,177].

Legume germination increases the amount of protein and dietary fiber, reduces the content of tannins and phytic acid, and improves the bioavailability of minerals [178]. During the germination process, proteolytic enzymes are activated, changing the protein profile of legumes and, consequently, of their derived beverages. The protein content in lentil-based beverages made of germinated seeds has been shown to be 3.3% higher (in terms of dry matter) than in a beverage made of non-germinated seeds. The germinated-lentil beverage contained more B vitamins and minerals compared to the beverage made from non-germinated lentils. This is attributed to the activity of α-amylase, which cleaves the high molecular weight carbohydrates that form cell membranes in plant materials. This leads to increased extractivity and thus more nutrients being transferred into the beverage [179]. In the soy-based beverage, the germination process increased the protein content while reducing fat, trypsin inhibitors, saponins, and phytic acid, with the added effect of inducing the proteolysis of the main storage proteins and releasing peptides that were easier to digest [152]. Compared to its non-germinated counterpart, the germinated bean-based beverage was characterized by higher “milk” yield, good color, and high sensory acceptability due to the lack of “beany” flavor and aroma [152]. 

Protein extraction is used to produce isolates or concentrates that not only have high protein content, but also better protein digestibility due to the elimination of ANFs [180]. Commercial protein isolates are usually obtained from legumes via alkaline protein extraction, which is followed by precipitating the extracted proteins at their isoelectric point [176,180]. To obtain legume-based beverages with greater stability, the formation of protein–polysaccharide conjugates can also be induced by spray drying and preparing a powder, which is then dissolved and reconstituted as a plant-based beverage [181].

The properties of the proteins may vary due to the use of processes such as high shear mixing, homogenization, and ultra-high temperature (UHT) processing in the production of legume-based beverages [174]. The associated pressure and thermal effects can lead to changes in the stability of protein emulsions [174,182]. Heat treatment, such as pasteurization, can increase the viscosity of the legume-based beverage, affecting its stability. This is important when the legumes are high in starch, such as chickpeas and peas [152]. Accordingly, additional processing techniques are also used to improve the stability of legume-based beverages. Advanced processes such as High Hydrostatic Pressure (HHP), Pulsed Electric Field (PEF), and Ultra High Pressure Homogenization (UHPH) have been successfully employed to improve the acceptability and properties of chickpea and faba bean beverages. These technologies are being rapidly developed thanks to ongoing research efforts, which could lead to the production of high-quality legume-based milk alternatives [21,173]. In addition to technological treatments, additives of various types of stabilizing substances are also used. The most common of those are hydrocolloids, which induce molecular interactions between the ingredients of the beverage, helping ensure a uniform consistency [183].

The sensory acceptability of legume-based beverages is the main limiting factor due to their characteristic “beany” flavor. This flavor is associated with the presence of endogenous lipoxygenases in the legumes, which oxidize unsaturated fatty acids [152,184]. Thermal inactivation is an effective technique for removing the “beany” flavor in legume-based beverages. The flavor has also been suppressed in a soy-based beverage by high-temperature (approx. 130 °C) steam treatment, or traditionally by boiling the beans prior to the grinding process, and finally by the germination process [152].

#### 5.2.1. Soy-Based Beverages

Among the legume-based beverages, the one most consumed and most widely available is derived from soy [152,173,185]. Soybeans are believed to be the first plant used in the preparation of plant-based beverage substitutes in China some 2000 years ago [21]. The soybean beverage has a high nutritional value and contains a similar amount of protein to cow’s milk (minimum 3%) [152]. Arnoldi et al. (2007) [186] report that soy proteins exhibit hypocholesterolemic properties. A soy-based beverage is an inexpensive, refreshing, and nutritious product with additional health-promoting ingredients, including isoflavones (such as genistein and daidzein) [20]. These ingredients are phytoestrogens, which have a chemical structure similar to that of estrodiol-17β, the most potent mammalian estrogen [187,188]. Isoflavones can help relieve postmenopausal symptoms and are well known for their protective effect against certain diseases such as hormone-dependent cancers (e.g., breast and endometrial cancer), cardiovascular disease, and osteoporosis [20,21,188,189]. 

Soybean beverages contain fiber, minerals (mainly iron, calcium, and zinc), B-group vitamins, unsaturated fatty acids, and plant sterols [20]. These products are also rich in phytochemicals such as phytosterols, which are known for their cholesterol-lowering properties [21]. Soy isoflavones elicit an antioxidant effect [187]. A study by Rossi et al. (2001) [187] showed that consumption of soybean beverage over 3 weeks (two servings per day providing 40 g protein and 44 mg genistein) increased the total plasma antioxidant values in each of the 10 male adolescents. 

The traditional basic method of producing a soybean beverage mainly consists of soaking, hulling cooking, wet grinding, filtering, and heat treatment to obtain the final product. The soybean beverage is similar in appearance to cow’s milk and is sold both sterilized and pasteurized, with or without flavoring [175]. 

Consumption of soybean beverages has been met with barriers in the form of consumer health concerns regarding genetic modification of soy, allergens in soy (mainly proteins β-conglycinin and glycinin), high levels of isoflavones, and trace CO_2_. Accordingly, it would be expedient to develop other milk alternatives from legumes—ones which may exhibit sensory properties similar or superior to those of soybeans [152]. The available products of this type which have been researched thus far include beverages made of peas, chickpeas, lupins, beans, and lentils.

#### 5.2.2. Pea-Based Beverages

Peas are a raw material that is widely available on a commercial scale [190]. Peas contain about 20–25% protein, which has properties similar to soybean protein and includes a large amount of the amino acid lysine. Compared to soybeans, peas have a higher level of dietary fiber, minerals, and vitamin C, as well as a lower fat content [190]. Pea proteins show promising functional properties, such as gelling or the ability to emulsify and foam across almost all pH ranges. What is more, these properties are retained after heat treatment [21,171]. Pea protein isolate is one of the most useful functional protein sources due to its high nutritional profile, high antioxidant potential, and low allergenicity compared to protein sourced from other plants [21]. These factors make peas a very good matrix for the production of plant-based beverages. Pea protein emulsions are more stable than other plant protein emulsions. However, the stability of pea-based beverages is highly dependent on the method used to process or prepare the beverage [21].

The nutritional value of peas varies depending on the degree of maturity [21]. For example, peas reach their highest concentration of sucrose, glucose, and fructose in the early stage of ripening, while these values decrease in fully ripe seeds with a concomitant increase in proteins and oligosaccharides. This trait makes it possible for manufacturers to pick and choose pea seeds with a specific degree of maturity, depending on the target nutritional value of the plant-based beverage [21].

Under laboratory conditions, a beverage was made from pigeon peas by first producing a powdered pea extract through the shelling, soaking, cooking, drying, and pulverizing of raw seeds [190]. The resultant beverage was shown to have hypoglycemic, hypocholesterolemic, and antioxidant properties in diabetic and hypercholesterolaemic rats. This indicates the potential of the pigeon pea-based beverage as a functional anti-diabetes product.

#### 5.2.3. Chickpea-Based Beverages

Chickpea is another legume that is a good source of protein and can be used to make plant-based beverages [171]. It is a good source of macro- and micronutrients, vitamins (such as thiamine and niacin), and minerals (such as magnesium, calcium, iron, and zinc) and is considered to be a suitable source of dietary protein due to its good balance of amino acids and high bioavailability [21]. Chickpea beverage is high in threonine, glycine, alanine, and arginine. Chickpea protein isolate can be used to make chickpea protein hydrolysates as it has good solubility and high protein quality compared to the protein found in raw seeds [171,184]. 

Though commercial chickpea beverages are available on the market, research into their properties is relatively limited [21]. Wang et al. (2018) [191] produced a garbanzo chickpea-based beverage by soaking, mixing, cooking, and filtering the liquid from its solid residues. Compared to soybean beverage, the chickpea beverage contained less protein, less fat, and more carbohydrates. Nevertheless, fresh chickpea beverage was determined to have potential as a substitute for soy-based beverages in terms of nutritional and organoleptic quality [191]. Other studies have also shown that the sensory acceptability of chickpea beverages was the same as that of soybean beverages [192].

#### 5.2.4. Lupine-Based Beverages

The main variety used in the production of lupine-based beverages is sweet lupine, grown on a large scale in Australia [82]. Lupine protein is a good source of arginine but has less sulfur-containing amino acids such as cysteine. Lupine contains mainly insoluble dietary fiber; however, its properties have been described as very similar to those of pectin and not to other insoluble non-starch polysaccharides. Its high carotenoid content gives lupine products a yellow color. Compared to its bitter cousins and other legumes, sweet lupine has negligible levels of anti-nutritional phytochemicals such as alkaloids, saponins, lectins, and phytates. Therefore, unlike most other legumes, sweet lupines do not require heating or chemical treatment to denature ANFs [82,173]. 

Lupine proteins have very good technological and functional properties, such as solubility and emulsification, which makes them a suitable raw material for the production of cow’s milk and soybean beverage substitutes [21]. Various methods have been developed to produce lupine-based beverages, but the basic process involves grinding the soaked lupine grain and mixing it with water to make a thick paste. This paste is then forced through a filter to obtain a milk substitute. However, this particular product has been shown to feature low stability, and some years later a beverage was produced based on a lupine protein extract obtained from lupine flour under alkaline conditions. The formulation was then diluted and blended with fat, carbohydrates, and bleaching agents, producing a lupine beverage with the desired organoleptic properties and nutritional value [21,186]. In addition to having favorable technological properties, sweet lupine also exhibits health-promoting attributes; it has hypoglycemic effects, induces satiety, promotes energy balance, and regulates the function of the circulatory and digestive systems [173,186].

#### 5.2.5. Bean-Based Beverages

Beans come in many varieties (including white, red, adzuki, and mung beans), but all of them are characterized by a high protein content, which is two to three times higher than in cereal grains. In addition, beans contain large amounts of dietary fiber, starch, vitamins, and minerals, as well as a wide range of phytochemicals [176]. Their glycemic index is low, not exceeding 27, so they are usually recommended for diabetic patients [20]. The basic bean-based beverage is prepared mainly by rinsing, soaking, grinding, and cooking the raw material. The obtained suspension is filtered and then thermally treated [20,172,193]. The resultant bean-based beverage contains the essential amino acids isoleucine, leucine, tyrosine, valine, asparagine, serine, glutamine, and proline [171]. Bean-based beverages are not produced on a large scale but have been successfully produced under laboratory conditions [193].

#### 5.2.6. Lentil-Based Beverages

Lentils are a valuable raw material that serves as a source of protein with a balanced amino acid composition and low fat content. It is also a valuable source of complex carbohydrates, soluble and insoluble fiber, vitamins, and minerals (including Na, Ca, Fe, P, and Cu) [171,179]. Lentil proteins have similar properties to soy proteins in terms of functionality and organoleptic properties [194]. Much like other legume-based drinks, lentil-based beverages are prepared by cleaning the grains, soaking the raw material, grinding, cooking, filtering, and heat-preserving [194]. Such products have not been produced on a commercial scale so far, but laboratory tests have been carried out to study their properties. A technology which has been used to successfully produce a lentil-based beverage harnesses the germination process, which increases its nutritional value and the protein digestibility of the final product [179]

#### 5.2.7. Legume-Based Beverages as Elements of Beverage Blends

Legume-based beverages are often used in research as elements of beverage blends, where they mainly serve to boost the protein content. The other ingredients are largely intended to improve the organoleptic properties and stability of the final product. 

Agrahar-Murugkar et al. (2020) [178] prepared beverages primarily composed of sorghum and finger millet (5.8% each), sprouting soy flour (1.1%), sprouting green-gram flour (0.7%), milk whitener (1.6%), and desiccated coconut (1.8%). In addition, one of the beverage variants contained jaggery (8.3%) and water (75%), whereas the other contained buttermilk (67%), cumin/black salt (1% each), and water (15%). Both variants were stable and rich in minerals, flavonoids, and antioxidants compared to most beverages on the market. Both products were acceptable to consumers. Sprouting legumes improved the solubility and nutrient extractability, while also increasing the level of antioxidants and flavonoids [178].

In a study by Rincon et al. (2020) [184], a new milk substitute based on chickpeas and coconut was developed, containing 70% chickpea extract, 30% coconut extract, and 0.3% vanilla extract. The beverage had beneficial nutritional composition (protein, calcium, and lipid content) compared to cow’s milk and other common cow’s milk substitutes such as oat, almond, and rice beverages. However, the sensory acceptance scores for the beverage were quite low [184].

Felberg et al. (2009) [175] developed a product based on a soybean beverage and Brazil nut beverage. The latter, with a dry matter concentration of about 10%, was added to soybean milk with a dry matter concentration of 7% at ratios ranging from 10 to 50% (based on the final beverage), after which 3% sugar and 0.2% salt were added. The addition of Brazil nut beverage to a soybean beverage positively influenced consumer response. The blend was more stable than the Brazil nut-based beverage alone [175].

Cereal proteins contain little lysine and tryptophan but provide optimal levels of sulfur amino acids (such as methionine and cysteine) [195]. The amino acid profile of legumes is rich in lysine but quite low in methionine and cysteine. Therefore, combining grains and legumes can improve the quality of the protein consumed [195]. Oladeji et al. (2014) [195] produced a beverage containing dried and ground preparations of sorghum and soybeans with the addition of cocoa powder and defatted melon flour. Cocoa powder, malted sorghum, soy flour, and skim melon flour were mixed to the ratio of 60:10:20:10 and 65:05:20:10. Thus prepared beverages had good physicochemical and organoleptic properties. It was also estimated that the production cost of such a beverage is low, which would make it a competitive product on the market [195].

#### 5.2.8. Other Potential Raw Materials for the Production of Legume-Based Beverages

Some legume seeds have yet to be used in the production of plant-based beverages, neither in commercial nor laboratory conditions. However, their properties make them potentially a good matrix for their production. These legumes include faba bean and cowpea. 

So far, faba bean protein has only been used to a limited extent on an industrial scale, but that is gradually changing. It is a good source of macro and micronutrients and minerals such as sodium, magnesium, calcium, iron, and zinc [21]. Faba bean is a protein-rich legume with similar properties to soybean proteins. Due to these properties, it can potentially be used as a soybean substitute in plant-based milk alternatives [174]. So far, to the best of the authors’ knowledge, faba bean-based beverages have not been commercialized, though there do exist faba bean concentrates with a favorable, neutral taste [21]. 

Cowpea is an important legume used mainly in East and West Africa, as well as in other developing countries. The total protein content of cowpea is approximately two to four times higher than that of tubers and cereals. In addition, cowpea protein is rich in amino acids such as lysine, phenylalanine, and histidine [21,171]. It is also a good source of bioactive and functional ingredients, such as phenols with antioxidant, anti-inflammatory, anti-cancer, hypolipidemic, and hypoglycemic effects. There are indications that any potential production of a cowpea-based beverage should best involve a sprouting process, as it significantly reduces the content of oligosaccharides [21].

### 5.3. Fermentation of Legume-Based Beverages Using LAB

An important direction in the development of plant-based beverages as milk substitutes is the use of fermentation in their production technology. Fermentation can lead to the production of a new range of products with a better sensory profile, nutritional properties, and improved microbiological safety [24,128,194,196]. Plant matrices are a good carrier for probiotic bacteria. Products made by plant fermentation with probiotics can meet consumers’ demands for health-promoting, dairy-free products [129,197,198,199,200]. Most of the plant-based beverages described in the literature are fermented using *Lactobacillus, Streptococcus*, and *Bifidobacterium* [24,129,194,201]. Fermentation of plant matrices is usually done through four main stages—obtaining a plant-based beverage; conditioning the beverage to reach the temperature optimal for the growth of the microorganisms; fermentation under specific conditions; and cooling to a temperature of about 4 °C [201]. These procedures may vary depending on the raw material, the type of the starter cultures used, and the final product characteristics. The fermentation of plant-based beverages is typically longer (around 12–24 h) than standard fermented dairy production and ends when the pH value is around 4.2–4.5 [21,201]. 

Fermentation of plant-based beverages significantly improves their nutritional and health-promoting properties. As in the fermentation of raw legumes, the content of oligosaccharides, tannins, protease inhibitors, and phytic acid is reduced, which increases the bioavailability of calcium, iron, and zinc [196,202,203,204]. In addition, the organic acids produced during fermentation have the ability to increase the absorption of iron and zinc by forming soluble ligands, while also producing a low pH that optimizes the activity of the endogenous phytic acid-reducing phytase [196,200]. Fermentation of legume-based beverages also increases antioxidant capacity and, as a result, anti-radical activity [200,205,206,207]. Some strains of LAB have the ability to synthesize B vitamins (including folic acid, riboflavin, and vitamin B12) in legume-based beverages, e.g., those made with soy and beans [31,208,209,210]. Microbial activity can also increase the content of minerals and protein in the product [196]. The use of the germination process before fermentation improves the growth rate of probiotic strains by increasing the content of fermentable monosaccharides and amino acids [127,193].

So far, out of all legume-based beverages, soybean beverages are the ones most commonly processed via fermentation. To that end, bacteria of the genus *Lactobacillus, Lactococcus*, and *Streptococcus* are generally used [211,212,213]. In order to select suitable LAB strains for the production of functional food based on a soybean beverage, the fermentation properties of 14 strains of LAB belonging to the genera *Bifidobacterium, Lactobacillus, Lactococcus*, and *Streptococcus* were assessed. All 14 strains were able to grow in the soybean beverage, and the strains *Bifidobacterium breve, Bifidobacterium bifidum*, and *Lacticaseibacillus rhamnosus* showed the most promising results [214]. Soy-based beverages fermented by LAB have a higher content of aglycone isoflavones. The increase of isoflavone aglycone contents during fermentation is a result of β-glucosidase activity towards isoflavone glucosides [205,212,215,216]. The use of LAB to ferment legume-based beverages can also have an anti-mutagenic effect. Fermentation of soy-based beverages using strains of the genera *Lactobacillus, Streptococcus*, and *Bifidobacterium* has been shown to significantly enhance anti-mutagenicity, but the extent of that increase varied across different starter organisms and types of mutagen tested [217].

Soymilk fermented by LAB can improve aroma, flavor, and overall acceptability [212]. Furthermore, they can be used to modulate and enhance the texture properties of soymilk, such as its water holding capacity and apparent viscosity [211,218]. Exopolysaccharide-producing (EPS-producing) LAB are used in the production of fermented soy beverages, due to their effect on consistency and rheology. Exopolysaccharides can modify the flow characteristics of fluids, stabilize suspensions, flocculate particles, encapsulate materials, and produce emulsions [211]. Fermentation of soybean beverages with LAB also reduces ANFs, e.g., fermentation with *Leuconostoc mesenteroides* removes phytates [203], whereas raffinose is removed during fermentation by bacteria of the genera *Lactobacillus* and *Streptococcus* [204]. LAB involved in soybean beverage fermentation may also exhibit anti-pathogen properties in food. Soybean beverage fermented with *Pediococcus pentosaceus* and *Lacticaseibacillus paracasei* subsp. *paracasei* demonstrated antimicrobial activity against selected foodborne pathogens, e.g., *Bacillus cereus, Staphylococcus aureus,* and *Pseudomonas aeruginosa* [219].

Bean, faba bean, lentil, chickpea, and cowpea beverages can also be good matrices for the fermentation process. Fermentation of a red bean-based beverage, using bacteria of the genera *Lactobacillus* and *Streptococcus,* has been shown to produce an increase in the total phenolic content and promote antioxidant activity [220], while fermentation of a navy bean-based beverage with *Lactobacillus* has been found to boost ACE-inhibitory activity [221]. An adzuki bean-based beverage fermented with *Lactococcus lactis* subsp. *lactis* and *Lacticaseibacillus rhamnosus* GG has shown increased levels of γ-aminobutyric acid (GABA) [222]. Fermentation of a bean-based beverage with the use of *Lactobacillus* strains can increase the share of palmitic, stearic, and oleic acids in the fatty acid profile compared to raw bean seeds [193]. Fermentation of a beverage made from germinated beans using yoghurt starter cultures Yo-Mix 205 LYO (*S. thermophilus, Lb. delbrueckii* subsp. *bulgaricus, Lb. acidophilus, B. lactis*) and FD-DVS ABY-3 Probio-Tec (*S. thermophilus, Lb. delbrueckii* subsp. *bulgaricus, Lb. acidophilus*, and *B. animalis* subsp. *lactis*) has been found to reduce stachyose and raffinose, while increasing riboflavin, niacin, and pyridoxine in the manufactured products [223]. 

Fermentation of a faba bean-based beverage using starter cultures containing bacteria from the genera *Lactobacillus, Lactococcus, Streptococcus*, and *Leuconocstoc* has been demonstrated to enhance the DPPH radical scavenging ability and total phenol content. The resultant beverage had higher complex viscosity values, which were expressed in a weak, gel-like structure [224]. A study by Verni et al. (2020) [194] showed that the strains of *Lactobacillus* spp., *Lb. helveticus, Lb. acidophilus, Lb. johnsonii, Lacticaseibacillus casei, Limosilactobacillus reuteri, Limosilactobacillus fermentum*, and *Lacticaseibacillus rhamnosus* were able to ferment a lentil-based beverage within 24 h, while strains *Lb. acidophilus, Limosilactobacillus fermentum*, and *Lacticaseibacillus paracasei* subsp. *paracasei* boasted the highest growth rates and the lowest pH values. The fermented beverages showed reduced levels of phytic acid and oligosaccharides [194]. 

A study by Wang et al. [191] demonstrated that a chickpea-based beverage can be a promising alternative to a soy-based beverage after some optimization. Compared to soymilk, the chickpea beverage contained lower amounts of protein, fat, and sugar, due to a higher starch content. Sensory analysis revealed that the fresh chickpea beverage was as acceptable as the soy one. However, the fermented chickpea beverage did receive lower appearance scores compared with the soy product [191]. A chickpea-based beverage fermented with *Lactiplantibacillus plantarum* subsp. *plantarum* has been demonstrated to have higher reducing power, and reduced content of β-conglycinin and glycinin, which are considered to be food allergens [225]. 

The cowpea-based beverage turned out to be a good fermentation matrix using probiotic cultures containing bacteria from the genera *Lactobacillus, Bifidobacterium*, and *Streptococcus* [198]. The obtained fermented beverage showed faster microbial growth during the first two weeks of storage. During this period, no significant differences were observed in terms of sensory attributes (taste, texture, and overall acceptability). However, the authors recommend that more work should be done to improve the sensory acceptability of the products, and that their potential health benefits should be determined through in vivo studies. The effect of fermentation with LAB on selected properties of legume-based beverages is summarized in Table 6.

## 6. Prebiotic Ingredients in Legumes and Legume-Based Beverages

Literature studies indicate that one of the most important determinants of human health is maintaining an optimal balance of the gastrointestinal microflora [226]. The relationship between gastrointestinal microflora and human health is being increasingly recognized. The influence of gastrointestinal microbiota on the host has been well characterized, including maintenance of the body’s energy metabolism and immune system [227]. Dysbiosis of the intestinal ecosystem can lead to certain illnesses, e.g., inflammatory bowel disease, irritable bowel syndrome, infectious and antibiotic-associated diarrhea, diabetes, and nonalcoholic fatty liver disease. These illnesses can be reversed by probiotics and prebiotics [228]. Probiotics can be defined as living bacteria or fungi that confer a health benefit for the host [228]. 

According to the International Scientific Association for Probiotics and Prebiotics (ISAPP), prebiotics are substrates that are selectively utilized by host microorganisms, eliciting health-beneficial effects [229]. The following criteria are used to classify a compound as a prebiotic:

- It should be resistant to acidic pH of stomach, cannot be hydrolyzed by mammalian enzymes, and should not be an absorber in the gastrointestinal tract;
- It can be fermented by intestinal microbiota;
- The growth and/or activity of the intestinal bacteria can be selectively stimulated by this compound, and this process is beneficial to the host’s health [230,231].

Prebiotics are not digested in the upper gastrointestinal tract and enter the cecum without changing their structure. They are not excreted in the feces as they are fermented by the flora of the colon, promoting the growth of beneficial bacteria from the genera *Bifidobacterium* and *Lactobacillus* [232,233,234]. During the fermentation, a mixture of short-chain fatty acids (SCFA) is produced, including acetate, propionate, and butyrate, as well as L-lactate, CO_2_, and H_2_ [233,235]. These compounds provide a broad range of important physiological benefits, including anti-inflammatory and immune regulation, as well as anti-cancer properties and metabolic regulation [235]. The alleged mechanisms of action of prebiotics may be direct or indirect. The indirect mode of action involves providing nutrients to the intestinal flora for natural growth, which is beneficial to health. A prebiotic may also act directly by inhibiting certain pathogenic bacteria, preventing cancer, removing cholesterol, controlling cardiovascular disease, and finally—preventing obesity and constipation [226].

Benefits of consuming prebiotics include improved intestinal barrier function and host immunity, reduction of potentially pathogenic bacterial subpopulations (e.g., *Salmonella typhimurium, Listeria monocytogenes, Escherichia coli*), and increased production of SCFAs [234], which helps regulate the absorption of sodium and water and may increase the absorption of calcium and other minerals. SCFAs lower the pH of the colon, which can inhibit the growth of potential pathogens and promote the growth of beneficial bacteria such as *Bifidobacterium* and *Lactobacillus* [226,234]. Propionate exerts an anti-inflammatory effect on colon cancer cells. Butyrate regulates apoptosis and reduces metastasis in colon cell lines. It is also the preferred energy source for colon epithelial cells, promotes normal cell differentiation and proliferation, and protects the body against carcinogens by enhancing the expression of enzymes involved in detoxification [226,234]. 

Legumes and legume-based beverages have proven to be a good source of food ingredients that may exhibit prebiotic properties [235,236,237]. These ingredients mainly include oligosaccharides, resistant starch, polyphenols, and isoflavones [237]. Legume oligosaccharides are often considered unbeneficial ingredients. Due to their high fermentability, they induce the production of gases (mainly CO_2_, H_2_, and sometimes CH_4_) responsible for the digestive discomfort associated with the consumption of legumes. In addition, their consumption may reduce the absorption of some nutrients. While it may be desirable to remove these components from legumes, they also exhibit favorable prebiotic properties [232,233,235].

Among the oligosaccharides of legumes with prebiotic properties, the most important are those of the raffinose family (RFO), also called α-galactosides [232,238]. They are low molecular weight, non-reducing carbohydrates that are widespread in the plant kingdom and soluble in water and hydroalcoholic solutions [238]. Chemically, α-galactosides are considered to be derivatives of sucrose, as they are a combination of d-galactose units linked to a group of d-glucose moieties. The oligosaccharides most common in legumes are raffinose, stachyose, and verbascose [192,232,238]. These compounds are not digested by the human gastrointestinal tract, as it does not produce α-galactosidase—an enzyme that has the ability to digest oligosaccharides by cleaving α-galactosyl moieties. As a consequence, these compounds are not broken down by digestive enzymes, so they are not absorbed in the upper part of the gastrointestinal tract and pass into the large intestine, where they promote the growth of bacteria from the genera *Bifidobacterium* and *Lactobacillus* [238,239].

*Bifidobacterium* and *Lactobacillus* prevent the growth of exogenous pathogenic microorganisms and the excessive growth of native harmful microflora, resulting in the production of SCFAs (mainly acetic and lactic acid) [232]. The production of SCFAs and subsequent acidification of the colonic contents affect the availability of minerals. Lower pH leads to increased solubility of minerals, especially calcium and magnesium, which consequently increases their absorption [232]. SCFAs strengthen the intestinal barrier by inhibiting the growth of pathogens and the production of toxic elements [236]. Oligosaccharides can inhibit bacterial adhesion to the gastrointestinal wall and act as a repressor of virulence factors by inhibiting gene expression in enteropathogens. They can adhere to bacterial binding sites on the surface of enterocytes, blocking adhesion of pathogenic bacteria to intestinal epithelial cells [226]. In a study with rats, prebiotic oligosaccharides from red gram beans have been shown to be hypolipidemic [240]. The authors pointed out that the use of prebiotics can be a potential as a preventive measure for overweight and obesity in humans, and legume prebiotics could be tested as a new prebiotic product candidate for the consumer market.

Resistant starch (RS) is the total amount of starch and products of starch degradation resistant to digestion in the upper gastrointestinal tract [241]. RS is a linear a-1,4-d-glucan molecule, which is a fraction of starch that is resistant to digestion by human pancreatic amylase in the small intestine, thus reaching the colon unchanged. In the colon, RS is fermented by intestinal bacteria [231,242]. Legume RS plays a role in improving digestive health and meets the criteria for classification as a prebiotic [241]. It can be fermented by the human gastrointestinal microflora, providing a source of carbon and energy for bacteria present in the gastrointestinal anaerobic environment, thereby potentially altering the composition of the microflora and its metabolic activity. Fermentation of carbohydrates by anaerobic bacteria produces SCFAs, mainly composed of acetic, propionic, and butyric acids, which can lower gut pH [242].

Resistant starch has no calories and does not increase blood glucose levels, having physiological effects similar to those of dietary fiber [237,241]. Its prebiotic effect can be enhanced by combining it with other types of prebiotics with complementary kinetics, such as fructooligosaccharides (FOS). These prebiotics are characterized by different fermentation rates in the large intestine, so their combination may result in a more pronounced probiotic effect through synergy. This combined effect may provide greater health benefits to the host. Additionally, the combination of RS and inulin has been shown to elicit a synergistic effect on intestinal calcium and magnesium absorption in rats. Fermentation of these substrates in the large intestine to SCFAs is a major cause of the increase in mineral absorption [242].

Scientific reports increasingly report interactions between polyphenols and the gastrointestinal microbiota, recommending them as candidates for prebiotics [243]. A growing number of animal and in vitro models describe the interactions between polyphenols and the gastrointestinal microbiota and the resulting health benefits, some of which include protection against cancer, obesity, insulin resistance, hepatitis, sleep deprivation, and atherosclerosis [244]. Polyphenols are secondary metabolites of plants, characterized by aromatic rings containing one or more hydroxyl groups in their chemical structure. Phenolic compounds are the largest group of phytochemicals, comprising over 50,000 heterogeneous compounds [245]. Polyphenols found in all types of legumes are characterized by low bioavailability and extensive metabolism in the large intestine, which promotes interaction with intestinal microorganisms [246]. One mode of two-way interaction involves polyphenols modulating the intestinal microflora, with microorganisms modulating the activity of phenolic compounds in turn. This interaction can regulate the metabolism and bioavailability of the polyphenols, converting them into metabolites that can have various effects on the health of the host. Overall, polyphenols elicit antioxidant, anti-inflammatory, anti-obesity, anti-lipidemic, and anti-diabetic effects. The role of polyphenols in the diet is determined by their metabolic processes, their absorption, and bioavailability, which are also associated with the modulation of the intestinal microflora [246]. 

Although polyphenols are currently recognized as modulators of the composition of the intestinal microflora, there is still no conclusive evidence of their prebiotic effects [247]. The prebiotic activity of each polyphenol can be influenced by the food source and the chemical structure of the compound, as well as by individual differences in the composition of the intestinal microflora. Flavonoids, which belong to polyphenols, are consumed with food mainly in the form of glycosides, which makes it difficult for their absorption through the small intestine. Polyphenols can act as prebiotics by modulating the microbiome, promoting the colonization of beneficial gut microbes. By acting as probiotics, the gut microbes are capable of degrading glycosided polyphenols and producing simple phenolic metabolites. The glycosylated flavonoids may serve as the sole source of carbon and energy for certain microorganisms in the intestinal microbiota that preferentially ferment the sugars associated with the flavonoids. Polyphenols can act as prebiotics to promote the growth of beneficial gut microbes such as *Bifidobacterium* and *Lactobacillus* spp. [245,246].

Among the ingredients of legumes, isoflavones may also show prebiotic properties. The highest concentration of isoflavones is found in soybeans [248]. These compounds belong to the class of hormone-like diphenol phytoestrogens and are similar in structure to the female estrogen 17β estradiol. The most popular soy isoflavone is genistein, which is consumed in the form of an α-glycoside called genistin. Upon ingestion, genistin is hydrolyzed to the aglycone genistein by ß-glucosidases. Enzymes capable of carrying out this stage of deglycosylation are found on the brush border of the small intestine (lactase-floridine hydrolase) and in enterocytes (cytosolic b-glucosidases). In addition, several major groups of colon bacteria have ß-glucosidase activity, including *Lactobacillus* spp., *Bacteroides* spp., and *Bifidobacterium* spp. It is this ability of these intestinal bacteria to break down isoflavone glycosides that may be related to the prebiotic effect of isoflavones, which stimulate the growth of these microorganisms. Pharmacokinetic studies confirm that healthy adults absorb genistein quickly and efficiently. The bioavailability of genistin depends on deglycosylation by the intestinal bacteria [248]. Equol and O-desmethylangolensin are active metabolites produced by the action of colonic bacteria on soy isoflavones. These metabolites also have health benefits, such as estrogenic, antioxidant, anti-inflammatory, antioxidant, and hepatoprotective effects. [246].

The prebiotic properties of legume ingredients make them a good matrix for the fermentation process, also with the participation of probiotic bacteria [226]. Both pre- and probiotics have been found to work best in combination. This combined effect of both leads to the formation of the so-called synbiotics. Prebiotic foods remain unchanged in the digestive tract because gastric enzymes cannot act on them. They reach the large intestine intact and are selectively fermented for beneficial effects [226]. Probiotics present in legumes may increase the survival rate of probiotic bacteria involved in the fermentation process, which pass through the upper part of the digestive tract after ingestion with food. This is the result of their selective stimulation by prebiotics. Prebiotics can also enhance the effects of probiotic bacteria that enter the large intestine [242].

## 7. Conclusions

LAB are widely used in the fermentation of animal and plant products. The most common use of LAB in food is in dairy production. Due to the growing popularity of plant diets and the health benefits of consuming fermented products, there has been growing interest in the fermentation of plant products and the selection of microorganisms suitable for this process. Research into microorganisms suitable for fermenting plant matrices could lead to an increase in the range of fermented plant products that can be used as alternatives to dairy.

Legumes are a suitable raw material for the production of dairy alternatives. This is mainly due to the high protein content and the presence of ingredients that enable their fermentation. Currently, legume fermentation is widely used in the production of intermediate bakery products (e.g., cookies, pasta, bread), ingredients for Asian cuisine, substitutes for dairy products, and as an ingredient of animal feed. The fermentation has also been found to elicit a beneficial effect on the bioconservation of legumes and their sensory properties. Fermentation reduces the content of thermostable ANFs in legumes, which are the main limitation in the development and consumption of legume-based foods. This process leads directly to an increase in digestibility of proteins and bioavailability of certain nutrients. Fermentation leads to an increase in the nutritional value of legume-based foods by increasing the content of antioxidant components, biological availability of total fiber and phenols, as well as increasing the levels of vitamins, amino acids, minerals, and short-chain fatty acids.

Legumes are a suitable matrix for the production of plant-based beverages, which are the most popular products among dairy alternatives. Among the legume-based beverages, soybeans are the most commonly used to produce plant-based beverages. Scientific reports indicate that there are also other legumes highly suitable for the production of plant-based beverages, e.g., beans, peas, broad beans, chickpeas, lupins, lentils, and cowpea. Creating beverages from legumes enables the production of plant-based beverages with a composition similar to cow’s milk. These products can be successfully fermented with LAB, including, e.g., *Lactobacillus delbrueckii* ssp. *bulgaricus, Lb. acidophilus, Lacticaseibacillus casei, Leuconostoc mesenteroides, Lactiplantibacillus plantarum* subsp. *plantarum, Lacticaseibacillus rhamnosus,* and *Streptococcus thermophilus*.

Both raw legumes and legume-based beverages can be carriers of probiotic bacteria. This is favored by the presence of natural ingredients with prebiotic properties in legumes, including oligosaccharides, resistant starch, polyphenols, and isoflavones. The properties of legumes make it possible to use them to create synbiotic food, which is a source of probiotics and prebiotics. However, in the production of fermented foods, it is crucial to optimize the process parameters. Too long fermentation time of legumes and legume-based beverages may affect the production of compounds with undesirable properties. The development of fermented products that can be commercialized requires careful development of technology and parameters of processing.

## Figures and Tables

**Table 1:** Healthy benefits of foods fermented with probiotic LAB.

Type of Food Product	Probiotic Microorganisms Used for Fermentation	Healthy Effects	References
Milk	*Lacticaseibacillus casei* Shirota	improvement of stool quality and frequency, lower water content in stools; increase in the intrinsic *Bifidobacteria*	Matsumoto et al., 2010 [52]
Dairy drink	*Lacticaseibacillus casei* DN-114 001	reduction of the average duration and of common infectious diseases	Guillemard et al., 2009 [53]
Milk	*Bifidobacterium breve* YIT 12272, *Lactococcus lactis* YIT 2027, *Streptococcus thermophilus* YIT 2021.	increase in the hydration levels of the stratum corneum, defecation frequency, feces quantity, and clearness of the skin	Mori et al., 2016 [54]
Milk	*Bifidobacterium animalis* subsp. *lactis, Streptococcus thermophilus, Lactobacillus delbrueckii* subsp. *bulgaricus, Lactococcus lactis* subsp. *lactis*	influence activity of brain regions that control central processing of emotion and sensation	Tillisch et al., 2013 [55]
Milk	*Lacticaseibacillus casei, L delbrueckii* subsp. *bulgaricus, Streptococcus thermophilus*	reduction in the incidence of antibiotic associated diarrhea and *Clostridium difficile* associated diarrhea	Hickson et al., 2007 [56]
Milk	*Lactobacillus helveticus*	reduction in the arterial stiffness in hypertensive subjects	Jauhiainen et al., 2010 [57]
Mung and adzuki bean sprouts	*Lactiplantibacillus plantarum* subsp. *plantarum* 299v	cytostatic and cytotoxic activity	Świeca et al., 2020 [58]
Soybean	*Lactiplantibacillus pentosus* var. *plantarum* C29	ameliorating effect against memory impairments	Yoo and Kim, 2015 [59]
Grape	*Lactiplantibacillus plantarum* subsp. *plantarum*	increase in the release and the intracellular content of inflammatory and anti-inflammatory cytokines; potential therapeutic measure to mitigating neuroinflammation in pathologies such as Parkinson’s disease and Alzheimer’s disease	Marzulli et al., 2012 [60]
Litchi juice	*Lacticaseibacillus casei* CICC 6117	enhance the immune organs indexes and the antioxidant capacity; increase in the secretions of cytokines and immunoglobulins; protection of the intestinal tract; alleviation of immune dysfunction and beneficial modification of gut microbiota structure	Wen et al., 2020 [61]

**Table 2:** The nutritional value of selected raw legumes, according to FoodData Central Search Results by U.S. Department of Agriculture [65].

Type of Legumes	Energy (kcal/100 g)	Protein (g/100 g)	Total Lipid (g/100 g)	Fatty Acids, Total Saturated (g/100 g)	Fatty Acids, Total Mono- and Polyunsaturated (g/100 g)	Carbohydrate (g/100 g)	Fiber (g/100 g)
Soybean	446.0	36.5	19.9	2.88	15.7	30.2	9.3
Bean, white	333.0	23.4	0.85	0.2	0.4	60.3	15.2
Bean, black	341.0	21.6	1.42	0.3	0.7	62.4	15.5
Bean, adzuki	329.0	19.9	0.53	0.2	0.2	62.9	12.7
Faba bean	341.0	26.1	1.53	0.2	0.9	58.3	25.0
Chickpea	378.0	20.5	6.0	0.6	4.1	63.0	12.2
Pea, pigeon	343.0	21.7	1.5	0,3	0.8	62.8	15.0
Lentil, red	358.0	23.9	2.2	0.4	1.6	63.1	10.8
Lupin	371.0	36.2	9.7	1.1	6.4	40.4	18.9
Cowpea, catjang	343.0	23.8	2.1	0.5	1.1	59.6	10.7

**Table 3:** The use of legumes fermented with LAB as ingredients of various types of food.

Type of Food	Type of Legume Used as A Food Ingredient	Microorganisms Used for Fermentation	Effect of the Addition of Fermented Legumes	References
Bread	Lupin	*Lactiplantibacillus plantarum* subsp. *plantarum* C48, *Levilactobacillus brevis* AM7	increase in the concentrations of free amino acids, soluble fiber, γ-aminobutyric acid (GABA) and total phenols; increase in the antioxidant activity	Curiel et al., 2015 [91]
Bread	Faba bean	*Weissella confusa* VTT E−143403 (E3403)	increase in the viscoelastic properties and specific volume; decrease in the crumb hardness	Wang et al., 2018 [92]
Bread	Chickpea	*Lactiplantibacillus plantarum* subsp. *plantarum*	decrease in the oligosaccharide content; increase in the free amino acids, lysine, and total phenolic content	Galli et al., 2019 [87]
Cookies	Lupin	*Latilactobacillus sakei* subsp. *sakei, Pediococcus pentosaceus, Pediococcus acidilactici*	decrease in the asparagine and sugar contents; decrease in the acrylamide content	Bartkiene et al., 2016 [93]
Pasta	Faba bean	*Lactiplantibacillus plantarum* subsp. *plantarum DPPMAB24W*	increase in the protein digestibility, nutritional indexes, and resistant starch content; decrease in the starch hydrolysis rate, without adversely affecting technological and sensory features	Rizzello et al., 2017 [94]
Tempeh	White bean	*Lactiplantibacillus plantarum* subsp. *plantarum* DSM 20174	increase in the protein, in vitro protein bioavailability, and antioxidant capacity; decrease in the stachyose, verbascose, and condensed tannins content	Starzyńska-Janiszewska et al., 2013 [95]
Ogi	Soybean	*Lactiplantibacillus plantarum* subsp. *plantarum*	increase in the protein, fat, iron and calcium content; decrease in the raffinose content; improvement of organoleptic attributes	Adeyemo and Onilude, 2018 [96]
Yogurt-style snack	Chickpea, lentil	*Lactiplantibacillus plantarum* subsp. *plantarum* DSM33326, *Levilactobacillus brevis* DSM33325	increase in the concentration of free amino acids and in vitro protein digestibility; decrease in the ANFs (i.e., phytic acid, condensed tannins, saponins and raffinose) content;	Pontonio et al., 2020 [97]
Camel milk and cow milk yogurt	Soybean	*Lactobacillus acidophilus LA-5, Bifidobacterium Bb-12, Lacticaseibacillus casei LC-01, Streptococcus thermophilus Th-4 and Lactobacillus delbrueckii* spp. *bulgaricus*	increase in the antioxidant activity; increase in the viability of LAB	Shori, 2013 [98]

**Table 4:** The effect of fermentation with LAB on selected properties of legumes.

Types of Legumes	Form of Raw Material	Microorganisms Used for Fermentation	Fermentation Conditions	Effect of Fermentation	References
Soybean	soybeans (*Glycine max L.)*	*Lactiplantibacillusplantarum* subsp. *plantarum* B1-6	37 °C, 30 h	reduction of the total saponin content, phytic acid content, and trypsin inhibitor activity; significant increase in the total phenolic content by the end of fermentation; improvement in the protein digestibility	Rui et al., 2017 [109]
	flour from soybeans (*Glycine max L.)* and fava beans (*Vicia faba L.*)	*Leuconostoc mesenteroides* subsp. *mesenteroides* DSM 20343	30 °C, 24 h	increase in the viscosity; decrease in the oligosaccharides content	Xu et al., 2017 [90]
	flour from soybeans (*Glycine max L. Merr*.)	(LAB)-consortium: *Lactiplantibacillus plantarum* subsp. *plantarum* WCFS1, *Levilactobacillus brevis* ATCC 14869, *Lacticaseibacillus rhamnosus* GG ATCC 53/03, *Companilactobacillus nantensis* LP33, *Limosilactobacillus fermentum* CIP 102980, *Limosilactobacillus reuteri* DSM 20016, *Pediococcus acidilactici* DSM 20284	spontaneous fermentation with 12 h intervals	decrease in the bulk density with increase in fermentation period; decrease in the swelling capacity; decrease in the later holding capacity; increase in the oil holding capacity; increase in the emulsion capacity	Ogodo et al., 2018 [89]
	soybean (*Glycine max L.) whey*	*Lactiplantibacillus plantarum* subsp. *plantarum* B1–6	37 °C, 24 h	increase in the total phenolic and isoflavone aglycone content; increase in the radical scavenging activity and protection against oxidative DNA damage	Xiao et al., 2015 [101]
	flour from yellow soybeans (*Glycine max* cv. *Merit*) and flour from Mung beans (*Vigna radiata*)	*Lactiplantibacillus plantarum* subsp. *plantarum* CECT 748 T	30 °C, 48 h	For fermented samples: increase in the bioactivity because of conversion of glycosylated isoflavones into bioactive aglycones; increase in the bioactive vitexin; increase in the tryptophan content	Landete et al., 2015 [103]
	soybeans (var. Rudoji and Progress) and wholemeal from lupin (*Lupinus luteus, L. albus*) seeds	*Latilactobacillus sakei* subsp. *sakei* KTU05-6, *Pediococcus acidilactici* KTU05-7, *P. pentosaceus* KTU05-8 (each strain applied separately)	30–35 °C (depending on the strain), 24 h	For all fermented samples: increase in protein digestibility; capacity to degrade phenylethylamine, spermine and spermidine by tested LAB strains; capacity to produce putrescine, histamine, and tyramine (biogenic amines) by tested LAB strains, but at levels lower than those causing adverse health effects	Bartkiene et al., 2015 [105]
Pea	pea (*Pisum sativum L*.) protein isolate	*Lactiplantibacillus plantarum* subsp. *plantarum* DSM-20174, *Lactobacillus perolens* DSM-12744, *Limosilactobacillus fermentum* DSM-20391, *Lacticaseibacillus casei* DSM- 20011, *Leuconostoc mesenteroides* subsp. *cremoris* DSM-20200, *Pediococcus pentosaceus* DSM-20336 (each strain applied separately)	30–37 °C (depending on the strain), 24 h and 48 h	For all fermented samples: after 24 h—aroma attributes and bitter taste decreased; after 48—cheesy aroma, acid and salty tastes were increased; decrease in the protein solubility and emulsifying capacity; foaming capacity remained constant; reduction in the intensity of the allergenic protein fractions	Garcia-Artegoa et al., 2021 [99]
	pea (*Pisum sativum L*.) protein isolate	*Lactobacillus acidophilus* NCFM, *Lb. delbrueckii* subsp. *bulgaricus, Streptococcus thermophilus*, *Bifidobacterium lactis* HN019 (strains applied in combination) with one of the yeasts: *Kluyveromyces lactis, Kluyveromyces marxianus, Torulaspora delbrueckii*	30 °C, until reaching pH 4.55	For all fermented samples: degradation of most of the molecules responsible for the leguminous and green off-notes; the presence of yeasts triggered the generation of esters; decrease in the intensity of the leguminous and green perception compared to strains without yeasts	Youssef et al., 2020 [126]
	pea (*Pisum sativum L*.) protein isolate	*Lactiplantibacillus plantarum* subsp. *plantarum*	37 °C, 25 h	decrease in the water-soluble protein content; reduction in off-flavor; desirable changes in aroma profile (removing the part of aldehyde and ketone content)	Shi et al., 2021 [104]
	pea seeds (*Pisum sativum* var. *Bajka*)	*Lactiplantibacillusplantarum* subsp. *plantarum* 299v	3 h, 72 h, and 168 h at 22, 30, and 37 °C	ACE inhibitory activity after in vitro digestion of fermented seeds for all samples	Jakubczyk et al., 2013 [115]
	flour from grass pea (*Lathyrus sativus L*.)	*Lactiplantibacillus plantarum* subsp. *plantarum*	30 °C, 24 h	reduction in the level of trypsin inhibitors; elimination of inositol phosphates; increase in the amount of total phenolics; partial improvement of the antiradical activity (with DPPH• assay)	Starzyńska-Janiszewska et al., 2011 [108]
Lupin	lupine flour (*Lupinus angustifolius*)	*Lactobacillus delbrueckii* subsp. *bulgaricus, Streptococcus thermophilus* (strains applied in combination)	30 °C, 20 h	increase in the mushroom, soil, green, and nutty aroma characteristics; modification of the overall aroma characteristics and potential improvement of the consumer acceptability of lupine products	Kaczmarska et al., 2018 [125]
	flour from sweet lupin (*Lupinus* *angustifolius*)	*Lactiplantibacillus plantarum* subsp. *plantarum* K779, *Limosilactobacillus reuteri* K777 (strains applied in combination)	35 °C, 72 h	noticeable antiproliferative activities against Caco-2 and MCF-7 cancer cell lines; pronounced antihypertensive activities; α-glucosidase inhibition; increase in the antioxidant activities	Ayyash et al., 2019 [116]
	lupin (*Lupinus angustifolius L*.) protein isolate treated with papain, alcalase 2.4 L, and pepsin	*Latilactobacillus sakei* ssp. *carnosus* DSM 15831, *Lb. amylolyticus* TL 5, *Lb. helveticus* DSM 20075 (each strain applied separately)	37–42 °C (depending on the strain), 24 h	For all fermented samples: increase in the foaming activity while maintaining proper emulsification capacity as a result of the combination of enzymatic hydrolysis and fermentation; increase in the protein solubility at acidic conditions; combination of enzymatic hydrolysis and fermentation was effective in breaking down large polypeptides into low molecular weight peptides and degrading with it the major allergen Lup an 1 of lupin	Schlegel et al., 2021 [85]
	lupin seeds (*Lupinus luteus L.* and *Lupinus albus L*.)	*Latilactobacillus sakei* subsp. *sakei* KTU05-6, *Pediococcus acidilactici* KTU05-7, *Pediococcus pentosaceus* KTU05-8 (each strain applied separately)	30–35 °C (depending on the strain), 24 h	For all fermented samples: increase in the protein digestibility; increase in the total phenolic compounds content; increase in the antioxidant activity	Krunglevičiūtė et al., 2016 [119]
Bean	red beans (*Phaseolus vulgaris*)	*Lactobacillus delbrueckii* subsp. *bulgaricus, Bacillus subtilis*	30 °C, 120 h	increase in the concentration of antioxidant substances, including total phenolics, anthocyanin, flavonoids, vitamins C and E; nattokinase activity exhibition	Jhanet al., 2015 [117]
	flour from kidney beans (*Phaseolus vulgaris* var. Pinto)	*Lactiplantibacillus plantarum* subsp. *plantarum* CECT 748	37 °C, 96 h	exhibition of potential antihypertensive activity due to their large γ-aminobutyric acid (GABA) content; activity of angiotensin converting enzyme inhibitory (ACEI)	Limón et al., 2015 [114]
	Mung bean (*Vigna radiata*), kidney bean (*Phaseolus vulgaris L*.), and soybean (*Glycine max. L*.),	*Lactobacillus delbrueckii* subsp. *bulgaricus* EMCC1102, *Lb. acidophilus* EMCC1324, *Lacticaseibacillus casei* EMCC1643 (each strain applied separately)	37 °C, 72 h	For all fermented samples: decrease in the phytic acid content	Mohamed et al., 2011 [122]
	red kidney beans (*Phaseolus vulgaris L.)*	*Lactobacillus* *acidophilus, Bifidobacterium, Streptococcus thermophilus* (strains applied in combination)	42 °C, 96 h	increase in the protein digestibility; reduction of phytates, trypsin inhibitor activity (TIA), saponins, tannins, and raffinose oligosaccharides	Worku et al., 2017 [123]
Faba bean	flour from faba bean (*Vicia faba* cv. Kontu)	*Lactiplantibacillus plantarum* subsp. *plantarum* VTT E-133328	30 °C, 48 h	decrease in the vicine and convicine contents; reduction of trypsin inhibitor activity and condensed tannins; increase in the amount of free amino acids, especially of the essential amino acids and GABA (γ-aminobutyric acid); enhancement in the in vitro protein digestibility; decrease in the hydrolysis index	Coda et al., 2015 [124]
	faba bean seeds (*Vicia faba L.* var. White Winston)	*Lactiplantibacillus plantarum* subsp. *plantarum* 299v	3 h, 72 h, and 168 h at 22, 30, and 37 °C	For all samples: ACE-inhibitory activity; antiradical activity against ABTS·+; LOX inhibitory activity	Jakubczyk et al., 2019 [111]
Lentil	lentil seeds (*Lens culinaris* var. *castellana*)	*Lactiplantibacillusplantarum* subsp. *plantarum* CECT 748T	37 °C, 96 h	increase in the GABA content; increase in the antioxidant capacity and angiotensin I-converting enzyme inhibitory (ACEI) activities; increase in the total phenolic compounds	Torino et al., 2013 [112]
Chickpea	flour from chickpea (*Cicer arietinum*) seeds	*Pediococcus pentosaceus, P. acidilactici*	spontaneous fermentation at 37 °C, 24 h with back-slopping for 10 days	reduction in the concentrations of raffinose and stachyose; elimination of verbascose; reduction of phytic acid; increase in the total phenolic contents; higher water-holding capacity of sourdoughs	Xing et al., 2020 [120]

**Table 5:** The nutritional value of selected plant-based beverages.

Category of Plant-Based Beverages	Type of Plant-Based Beverages	Protein (g/100 g)	Total Lipid (g/100 g)	Carbohydrate (g/100 g)	Fiber (g/100 g)	References
Cereal-based beverages	Oat-based beverage	0.4–1.0	0.7–1.9	6.5–27.0	-	Mäkinen et al., 2015 [127]; Parrish 2018 [142]; Veber et al., 2021 [143]
	Rice-based beverage	0.1–0.8	0.9–2.6	9.1–27.0	0.0–0.1	Chalupa-Krebzdak et al., 2018 [144]; Mäkinen et al., 2015 [127]; Vanga and Raghavan, 2018 [145]
Legume-based beverages	Soy-based beverage	2.1–3.0	1.3–3.2	1.8–4.7	0.7–1.3	Manzoor et al., 2017 [146]; Shen et al., 2019 [147]; Giri and Mangaraj, 2012 [148]; Jiang et al., 2013 [149]
	Pea-based beverage	2.8–7.9	0.1–4.5	10.0–27.0	n.d.	Parrish 2018 [142]; Veber et al., 2021 [143]; Pandhi and Poonia 2021 [150]
	Lupin-based beverage	1.8–3.5	1.3–1.4	1.2–3.3	n.d.	Vogelsang-O’Dwyer et al., 2021 [151]; Lopes et al., 2020 [152]; Laaksonen et al., 2021 [86]
Nut-based beverages	Cashew-based beverage	0.4–2.2	1.0–5.2	3.7–5.7	0–1.1	Manzoor et al., 2017 [146]; Chalupa-Krebzdak et al., 2018 [144]; Singhal et al., 2017 [153];
	Almond-based beverage	0.3–2.1	0.8–4.4	0.2–3.3	1.0–1.6	Chalupa-Krebzdak et al., 2018 [144]; Jeske et al., 2017 [131]; Mäkinen et al., 2015 [127]; Vanga and Raghavan 2018 [145]
	Hazelnut-based beverage	2.3–4.5	1.5–6.5	2.3–3.2	-	Atalar 2019 [154]; Aysu et al., 2020 [155]; Gul et al., 2021 [156]
Seed-based beverages	Sesame-based beverage	2.6–2.9	6.4–7.8	4.0–16.5	0.0–0.5	Afaneh et al., 2011 [157]; Sethi et al., 2016 [19]; Ahmadian-Kouchaksaraei et al., 2014 [158]
	Hemp-based beverage	0.8–1.9	1.2–7.0	2.2–7.9	0.0–0.2	Chalupa-Krebzdak et al., 2018 [144]; Mäkinen et al., 2015 [127]; Parrish 2018 [142]
Pseudocereal-based beverages	Quinoa-based beverage	0.4–4.5	0.2–6.0	9.0–15.5	-	Kaur and Tanwar, 2015 [159]; Sethi et al., 2016 [19]; Pineli et al., 2015 [160]
	Buckwheat-based beverage	0.2–4.3	0.0–1.2	4.6–8.8	0.0–0.9	Cardinali et al., 2021 [161]; Kowalska and Ziarno 2020 [162]; Zhou et al., 2019 [163]
Other plant-based beverages	Coconut-based beverage	<1	3.2–5.0	0.7–30.1	0.0–1.0	Vanga and Raghavan, 2018 [145]; Sethi et al., 2016 [19]; Lu et al., 2019 [164]

n.d.—no data.

**Table 6:** Effect of fermentation with LAB on selected properties of legume-based beverages.

Type of Legume-Based Beverages	Ingredients of the Beverage	Microorganisms Used for Fermentation	Fermentation Conditions	Effect of Fermentation	References
Soy-based beverages	soybean seeds, water	*Lactobacillus acidophilus* ATCC^®^ 4356™, *Lacticaseibacillus casei* ATCC^®^ 393™ (strains applied as a mixed cultures)	37 °C, 12 h	increase in the viscosity; increase in the antioxidant activity; increase in the isoflavones (genistein and daidzein) content; improvement of the sensory evaluation for parameters: color, texture, aroma, flavor, overall acceptability	Ahsan et al., 2020 [212]
	soybean seeds, water, sucrose,	*Lactiplantibacillus plantarum* subsp. *plantarum* 70810, *Lacticaseibacillus rhamnosus* 6005, yogurt starter culture DVS YC-X11 (*Lb. delbrueckii* subsp. *bulgaricus, Streptococcus thermophilus*) (strains applied separately)	37 °C, 12 h	increase in the water holding capacity, apparent viscosity, and exopolysaccharide (EPS) amount (highest with *Lactiplantibacillus plantarum* subsp. *plantarum* 70810); EPS-protein improved the texture of fermented beverage; increase in the concentration of the characteristic flavor compounds and decrease in the beany off-flavor (investigated only for *Lactiplantibacillus plantarum* subsp. *plantarum* 70810)	Li et al., 2014 [211]
	soybean seeds, water	*Leuconostoc mesenteroides* KC51	30 °C, 18 h	decrease in the phytate content	Oh et al., 2009 [203]
	soybean seeds, water	*Lactobacillus helveticus* R0052, *Bifidobacterium longum* R0175, *Streptococcus thermophilus* ST5 (strains applied single or as a mixed cultures)	30 °C, until pH 4.7 was reached	decrease in the isoflavones level with *L. helveticus* R0052 and combination of *S. thermophilus* ST5 + *L. helveticus* R0052; fermentation did not significantly modify vitamin B1 or B6 levels	Champagne et al., 2010 [218]
	commercial soymilk	*Lactobacillus acidophilus* CCRC 14079, *Streptococcus thermophilus* CCRC 14085, *Bifidobacterium infantis* CCRC 14633, *B. longum* B6 (strains applied single or as a mixed cultures)	37 °C, 24–32 h (depending on the strain)	major reduction in the contents of glucoside, malonylglucoside, and acetylglucoside isoflavones along with a significant increase of aglycone isoflavones content	Chien et al., 2006 [215]
	commercial soymilk, yeast extract, glucose	*Lactobacillus acidophilus* LAFTI L10, *Lb. delbrueckii* ssp. *bulgaricus* Lb1466, *Lb. acidophilus* La4962, *Lacticaseibacillus casei* LAFTI L26, *Lacticaseibacillus casei* Lc279, *Bifidobacterium lactis* LAFTI B94, *B. longum* Bl 536, *Streptococcus thermophilus* St1342 (strains applied as a mixed culture)	42 °C, 48	reduction of raffinose content; release of bioactive peptides with ACE-inhibitory activities	Donkor et al., 2007 [204]
	soybean seeds, water	*Lactobacillus acidophilus* CCRC 14079, *Streptococcus thermophilus* CCRC 14085, *Bifidobacterium infantis* CCRC 14633, *B. longum* B6 (strains applied single or as a mixed cultures)	37 °C, 32 h	fermentation significantly enhanced the antimutagenicity of soymilk (the levels of increased antimutagenicity of fermented soymilk varied with the starter organism and the type of mutagen tested)	Hsieh et al., 2006 [217]
Bean-based beverages	navy bean seeds, water	*Lactiplantibacillus plantarum* subsp. *plantarum* B1-6, *Lactiplantibacillus plantarum* subsp. *plantarum* 70810, *Lb. delbrueckii* subsp. *bulgaricus, Lb. helveticus* MB2-1 (strains applied separately)	31–42 °C (depending on the strain), 6 h	increase in the ACE inhibitory activity; decrease in the protein content with *Lb. delbrueckii* subsp. *bulgaricus* and *Lactiplantibacillus plantarum* subsp. *plantarum* B1-6	Rui et al., 2015 [221]
	red bean powder, water, refined sugar	*Lacticaseibacillus casei* 388, *Lactiplantibacillus plantarum* subsp. *plantarum* 299v, *Streptococcus thermophilus* TISTR 894 (strains applied single or as mixed cultures)	37 °C, 18–20 h	increase in the total phenolic contents and antioxidant activities	Naprasert et al., 2019 [220]
	white bean seeds, water	industrial starter cultures: Yo-Mix 205 LYO (*Streptococcus thermophilus, Lactobacillus delbrueckii* subsp. *bulgaricus, Lb. acidophilus, Bifidobacterium lactis*) and FD-DVS ABY-3 Probio-Tec (*S. thermophilus, Lb. delbrueckii subsp.bulgaricus, Lb. acidophilus* La-5, *B. animalis* subsp. *lactis* BB-12) (cultures applied separately)	43 °C, 4 h	increase in the content of stachyose and raffinose; increase in the levels of riboflavin, niacin, and pyridoxine	Ziarno et al., 2019 [223]
	adzuki bean flour, water	*Lactococcus lactis* subsp. *lactis, Lacticaseibacillus rhamnosus* GG (strains applied as a mixed cultures)	37 °C, 24 h	increase in the content of γ-aminobutyric acid (GABA)	Liao et al., 2013 [222]
	white bean seeds, water	*Lactobacillus delbrueckii* subsp. *bulgaricus* ATCC 11842, *Lb. delbrueckii* subsp. *lactis* ATCC 4797, *Lb. acidophilus* La3, *Lb. helveticus* LH-B01, *Lactiplantibacillus plantarum* subsp. *plantarum* DSM 9843, *Lacticaseibacillus rhamnosus* LH32, *Limosilactobacillus fermentum* ATCC 9338, *Levilactobacillus brevis* L342, *Lacticaseibacillus casei* 01, *Lacticaseibacillus paracasei* subsp. *paracasei* BGP1	37 °C, 18 h	increase in the share of palmitic, stearic, and oleic acids in the fatty acid profile compared to that in raw bean seeds; lower share of palmitic and stearic acids and higher share of oleic acid in position sn-2 was observed compared to non-fermented beverages	Ziarno et al., 2020 [193]
Faba bean-based beverages	faba bean and chickpea seed, water	Starter culture 1 contains *Streptococcus thermophilus* and *Lactobacillus delbrueckii* subsp. *Bulgaricus*, and starter culture 2 contains *Lacticaseibacillus casei, Lactococcus lactis* subsp. *cremoris, Lc. lactis* subsp. *lactis, Lc. lactis* subsp. *lactis bio* var. *diacetylactis, Leuconostoc species, Streptococcus thermophilus* (starters applied separately)	43 °C, 10 h	increase of the DPPH radical scavenging ability and total phenol content; higher complex viscosity values for faba bean-based products, which displayed a weak gel-like structure	Vasilean et al., 2021 [224]
Lentil-based beverages	lentil seeds, water	*Lactobacillus acidophilus* ATCC 4356, *Lb. gasseri* ITEM 13541, *Lb. helveticus* ATCC 15009, *Lb. johnsonii* NCC533, *Lacticaseibacillus rhamnosus* ATCC 53103, *Lacticaseibacillus paracasei* subsp. *paracasei* DSM 20312, *Limosilactobacillus fermentum* DSM 20052 (strains applied separately)	37 °C, 24 h	decrease in the phytic acid and raffinose content	Verni et al., 2020 [194]
Chickpea-based beverages	chickpea seeds, water, with addition of soy sauce or vanillin sugar and coconut flakes	*Lactiplantibacillus plantarum* subsp. plantarum 299v	35 °C, 18 h	increase in the reducing power; decrease in the content of β-conglycinin and glycinin (which are considered as one of food allergens)	Skrzypczak et al., 2019 [225]
	garbanzo chickpea seeds, water	*Streptococcus thermophilus, Lactobacillus delbrueckii* subsp. *bulgaricus, Lb. acidophilus*	42 °C, 16 h	lower amounts of protein, fat, and sugar, and higher starch content compared to soymilk; fermented chickpea beverage received lower ratings than the soy one for appearance	Wang et al., 2018 [191]
Cowpea	cowpea seeds, water	Probiotic starter cultures: ABT-5 *(Lactobacillus acidophilus La-5 + Bifidobacterium animalis* Bp-12 *+ Streptococcus thermophilus),* YFL-903 *(S. thermophilus + Lb. bulgaricus subs. debulgaricus)* and Yoba Fiti *(Lacticaseibacillus rhamnosus GR-1 + S. thermophilus)* (cultures applied separately)	45 °C, 14 h	decrease in the carbohydrate content; increase in microbial growth during the first two weeks of storage	Aduol et al., 2020 [198]
